# Emerging Nanotherapeutic Approaches to Overcome Drug Resistance in Cancers with Update on Clinical Trials

**DOI:** 10.3390/pharmaceutics14040866

**Published:** 2022-04-15

**Authors:** Syed Nasir Abbas Bukhari

**Affiliations:** Department of Pharmaceutical Chemistry, College of Pharmacy, Jouf University, Sakaka 72388, Saudi Arabia; sbukhari@ju.edu.sa; Tel.: +966-565738896

**Keywords:** cancer, nanotherapeutics, cancer stem cells, drug resistance, tumor microenvironment

## Abstract

A key issue with modern cancer treatments is the emergence of resistance to conventional chemotherapy and molecularly targeted medicines. Cancer nanotherapeutics were created in order to overcome the inherent limitations of traditional chemotherapeutics. Over the last few decades, cancer nanotherapeutics provided unparalleled opportunities to understand and overcome drug resistance through clinical assessment of rationally designed nanoparticulate delivery systems. In this context, various design strategies such as passive targeting, active targeting, nano-drug, and multimodal nano-drug combination therapy provided effective cancer treatment. Even though cancer nanotherapy has made great technological progress, tumor biology complexity and heterogeneity and a lack of comprehensive knowledge of nano-bio interactions remain important roadblocks to future clinical translation and commercialization. The current developments and advancements in cancer nanotherapeutics employing a wide variety of nanomaterial-based platforms to overcome cancer treatment resistance are discussed in this article. There is also a review of various nanotherapeutics-based approaches to cancer therapy, including targeting strategies for the tumor microenvironment and its components, advanced delivery systems for specific targeting of cancer stem cells (CSC), as well as exosomes for delivery strategies, and an update on clinical trials. Finally, challenges and the future perspective of the cancer nanotherapeutics to reverse cancer drug resistance are discussed.

## 1. Introduction

In spite of recent technical and pharmacological advances, cancer continues to be one of the top causes of human death globally, accounting for around 13% of all fatalities each year aldehyde dehydrogenase [1]. Approximately 21 million more cases and a 13 million increase in cancer-related deaths are projected by 2030, according to the National Cancer Institute (NCI). A 50% rise in diagnosed cases and a 60% increase in cancer-related fatalities by 2030 is predicted compared to 2012, when there were only 14 million cases and 8.2 million deaths [2]. For many decades, surgery, radiation therapy, and chemotherapy were the most popular cancer treatment options. The fact that these therapeutic procedures are still regularly utilized in conjunction with other specialist strategies for the treatment of cancer should be noted. Surgery is particularly beneficial for solid tumors and for cancers that have not spread to other parts of the body. After surgical excision of solid tumors, radio- and/or chemoradiotherapy is used to overcome the constraints of surgery [3]. For the majority of cancer patients, both radiation and chemotherapy are used as a primary therapeutic option.

**Figure 1:**
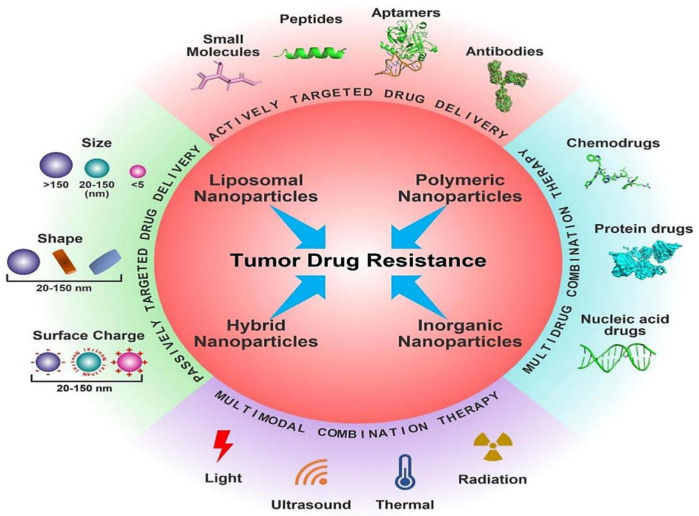
Different nanotherapeutic approaches for overcoming cancer drug resistance. Reproduced from Ref. [12], (2022), with permission from Elsevier.

Conventional chemotherapy remains a powerful tool and curative measure in eliminating malignant cancer, yet there is a pertinent need for development of alternative treatment modalities due to certain limitations [4]. These limitations include tumor heterogeneity, clonal evolution, transcriptional mutations, multi-drug resistance (MDR), and systemic toxicities which limits the efficacy of several drugs in clinical settings which showed proven antitumor properties in preclinical studies [5]. In order to overcome these limitations, various modified therapeutic approaches such as palliative care, use of targeted biological agents and other approaches were employed to optimize the effect of conventional therapies [6]. However, a steep increase in overall cancer burden limits the therapeutic modalities especially due to cancer drug resistance or non-responsive characteristics towards therapy [7]. Moreover, emergence of multi-drug resistance (MDR) an insurmountable hindrance in chemotherapy and ultimately compromise the cancer therapy [8,9,10,11]. Some of the ways that cancer is resistant to drugs are caused by changes in the tumor microenvironment, the number of different types of cancer cells in a given area, the ability of cancer cells to take up the drugs, and the ability to get rid of drugs that are not taking them out of their bodies [12]. Cancer stem cells CSCs) were considered as important sources of drug resistance in the past few years owing to its intrinsic unique properties [11,13,14,15]. CSCs are usually in quiescent phase and escapes the chemotherapy and radiotherapy treatments which further develops drug resistance [6]. The unique and specific properties of CSCs include self-renewal ability, cell proliferation and differentiation ability, molecular plasticity, expression of specific surface markers, DNA repairability, hypoxic stability, increased expression of ATP binding cassette (ABC) transporters, and antiapoptotic protein overexpression, contributing to drug resistance [16,17]. Therefore, there is a pressing need for development of improved therapeutic regimens for cancer therapy in order to overcome cancer drug resistance. In the past few years, multiple treatment strategies such as anti-angiogenesis therapy, immunotherapy, target therapy, nanotherapy, signal modification therapy, apoptosis regulations, nucleic acid-based therapies, and other therapies have escalated much attention for regulating immune function, inhibition of cell proliferation, limiting angiogenesis and metastasis, induction of apoptosis and reversal of MDR [18,19,20,21,22,23]. Among all these treatment approaches, nanotherapeutics-based approaches hold great potential for overcoming the limitations of conventional therapies for cancer therapeutics and diagnosis due to their multifunctional potential. Moreover, cancer nanotherapeutics are swiftly progressing and perhaps it is most explored therapeutic option. The unique physicochemical properties of nanotherapeutics allow them to overcome some of the drawbacks of traditional medicines, such as short half-life, poor water solubility, poor oral bioavailability, and non-specific biodistribution. Better bioavailability, increased pharmacokinetics and improved targeted drug delivery and tumor penetration are some of the many advantages that nanoparticle-based drug carriers provide in cancer therapy. They also have fewer side effects and may be used to treat a wide range of cancers [24]. The plasticity of nanoparticle composition and surface chemistry promotes a wide range of design options. As a result, a nano-drug-based delivery system not only delivers targeted drug administration to overcome drug resistance, but also specificity to cancer cells and diagnostics. Nanotherapeutics approaches using different kinds of nanocarriers for overcoming drug resistance are shown in Figure 1.

**Figure 2:**
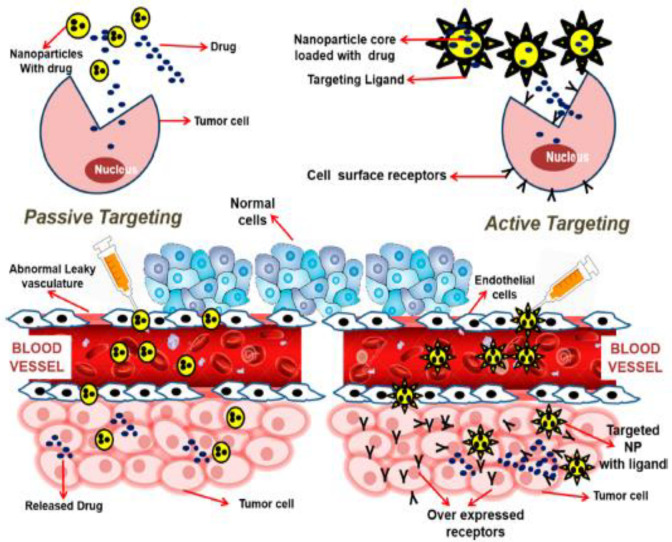
Active and passive targeting approaches in cancer nanotherapeutics. Reproduced from Ref. [32], (2022), with permission from American Chemical Society (ACS).

Nanocarriers provide extraordinary specificity in terms of targeted delivery through both active and passive targeting mechanisms, as shown in Figure 2 [25]. In passive targeting, nanocarriers employs enhanced permeability and retention (EPR) effect which is promoted by abnormal leaky vasculature and lack of lymphatic drainage in tumor microenvironment. As a result, extravasation within tumor tissues and increased accumulation of therapeutic agents at tumoral site are facilitated [26]. However, specific targeting inside the tumor site is challenging using passive targeting approach as heathy tissues can get affected too with this approach and compromises the drug biotherapeutic window. In active targeting, nanocarriers are conjugated with tumor specific ligands that can interact with overexpressed surface receptors on target cells with reduced toxicity. The biocompatible targeting ligands used in active targeting include antibodies, aptamers, peptides which are specific to receptors, or antigens at the tumor site [27,28].

Overall, this approach provides a plethora of advantages, including targeted specific delivery, increased biodistribution and therapeutic window, reduced non-specific toxicities, less immunogenicity, and systemic circulation [29,30]. Furthermore, active targeting may utilize combination therapy by taking advantage of the synergistic effects of different drugs/therapeutic agents for delivery of multiple therapeutic agents, such as imaging and/or theranostic agent for multimodal functions [31].

Recent advancements in nanotherapeutics fueled the development and exploration of various nano-based vehicles for efficient drug/therapeutic agents’ delivery. Some of the commonly utilized nano vehicles include lipid- and micelle-based nanoparticles, polymeric/non-polymeric nanoparticles, nanoconjugates, carbon nanotubes, nanogels, nano capsules, dendrimers, polymer micelles, and quantum dots for the enhancement of the efficacy of therapeutic interventions by conveying vast payload without toxicity [12,33,34]. In the past few years, numerous nanoformulations were widely investigated and used for cancer imaging and diagnosis. However, in order to realize the cancer nanotherapeutic potential, nanoparticle-based delivery systems must overcome a number of obstacles and biological barriers before reaching the target tumor location [35].

Currently, advancements in materials science and protein engineering paved the way for the design and development of newer and innovative nanoscale targeting strategies for cancer therapy. To attempt this, various nanoformulations-based platforms, such as albumin nanoparticles and liposomes, were approved for clinical use [36]. Several other nanotechnologies-based therapeutic modalities are under clinical investigation [37]. The FDA-approved nanoparticles-based delivery systems, including Aroplatin Abraxane^®^, doxorubicin-loaded liposomes, paclitaxel-bound albumin, OSI-211, and Oncaspar^®^, have significant anticancer activity [38]. Additionally, many nanomaterials-based systems, such as Aurimune, CRLX101/Camptothecin, Lipoplatin, AuroShell, nd 30 plus nanoconjugates, still remain to be tested through different clinical trials [39].

In this review, an overview of cancer nanotherapeutics and its advancements is provided. Herein, I highlight the current chemotherapeutics open challenge, cancer drug resistance, its mechanisms, and need for cancer nanotherapeutics. Furthermore, I specifically review different emerging and innovative nanotherapeutics-based strategies for cancer therapy, namely, strategies for targeting tumor microenvironment (TME) and its components, noncoding RNA-based targeting (siRNA and miRNA-based delivery systems), exosomes-based targeting strategies, self-assembly prodrug-based strategies, and advanced delivery systems for cancer stem cells (CSCs) and integrin targeting followed by detailed update on clinical trials status. Finally, I discuss the current challenges and future prospective of cancer nanotherapeutics.

## 2. Drug Resistance in Cancers and Its Mechanisms

**Figure 3:**
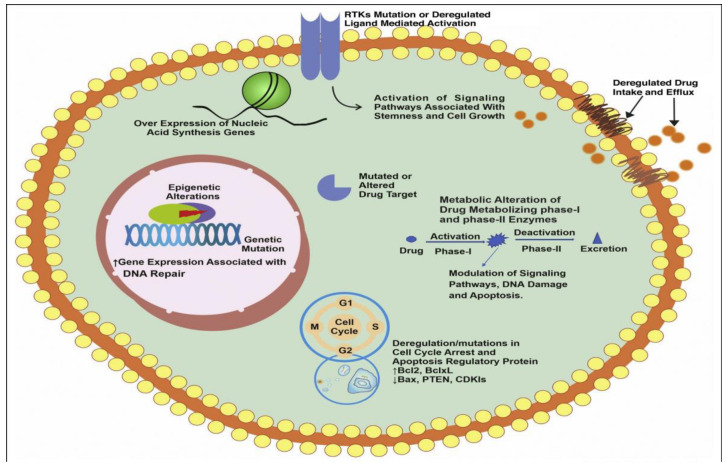
Different underlying mechanisms associated with drug resistance. This figure shows different intrinsic and extrinsic factors responsible for cancer drug resistance starting from alteration of signaling pathways, remodeling of drug efflux pumps expression, overexpression of genes related to cell cycle and apoptosis, enhanced expression of nucleic acid synthesis genes, enhanced DNA repair ability, alteration of drug target sites, alteration in functioning of drug metabolizing enzymes, genetic alternations, and epigenetics. Reproduced from Ref. [40], (2022), with permission from Elsevier.

Chemotherapy represents one of the principal modes for treatment of cancer. However, the development of MDR has become a major problem in oncology and limits the effectiveness of chemotherapy for the treatment of different metastatic cancers. Multi-drug resistance highlights the resistance to multiple different drugs which are structurally and functionally distinct from the original drug. Accumulating evidence indicate resistance to cancer therapeutics is a complex and challenging process and requires considerable and immediate attention along with rigorous understanding of the underlying mechanisms [40]. As per available findings, drug resistance could be defined into either intrinsic resistance or extrinsic resistance based on the factors associated with it. According to the cancer type, drug resistance might be inherited or acquired. These two types of medication resistance (extrinsic vs. intrinsic and inherited vs acquired) cause the doctors a significant therapeutic problem. This type of resistance emerges as a result of the existence of resistance-mediating elements in cancer cells and their environment. Extrinsic or acquired drug resistance, on the other hand, may emerge during the treatment of tumors that were previously responsive to cytotoxic medicines. Extrinsic resistance would compensate for the therapeutic effects of previously used drugs, and this could occur as a result of a variety of adaptive responses, including the modulation of signaling pathways, activation of alternative signaling pathways, and increased expression of the therapeutic target [41]. Moreover, the modulation of signaling pathways is responsible for the regulation and reprogramming of different metabolic and cellular physiological pathways, tumor microenvironment, stemness, and cancer resistance. Among other factors, proinflammatory cytokines, chemokines, and reactive oxygen species (ROS) play vital roles in the modulation of different signaling pathways. Overall, different factors, causes, and mechanisms which are associated with the drug resistance in different types of cancers (combining both extrinsic and intrinsic resistance) include change in tumor microenvironment, tumor heterogeneity due to cellular changes, reduced drug uptake, inactivation of drug, alteration of drug targets, drug efflux, cell death inhibition, alternation in DNA repair process, epigenetics, inhibition in apoptotic pathways and autophagy, epithelial to mesenchymal transition, metastasis, and many more as discussed in [17,42,43]. Therefore, it is pertinent to understand cancer resistance phenomenon and the cardinal signaling mechanisms arising from various exogenous and endogenous factors towards development of future therapeutic interventions or combination therapy for different cancers. A list of different factors which induce drug resistance in cancer are presented in Figure 3 and Table 1.

In following sections, we discuss some major causes/factors leading to drug resistance in cancer chemotherapy. Tumor microenvironment (TME) is considered one of the vital factors for development of drug resistance during cancer treatment in some cancers as a result of interactions between the cancer cells and adjacent TME components [57]. The tumor microenvironment comprises of cellular and non-cellular components and their interactions induces cancer drug resistance and putting therapeutic pressures in clinical settings. The cellular components of TME include cancer-associated fibroblasts (CAFs), myeloid, lymphoid, endothelial, and stromal cells, while non-cellular components comprise of soluble factors such as cytokines, chemokines, various growth factors such as vascular endothelial growth factor (VEGF), fibroblast growth factors (FGFs), insulin-like growth factors (IGFs), platelet-derived growth factor (PGDF), B-cell activating factors, and others. For example, the development of anti-VEGF/VEGFR drugs resistance in renal cell carcinoma treatment is caused by the production of pro-angiogenic factors, such as FGFs, PDGFs, etc. [58]. In another report, disease progression and drug resistance in metastatic colon cancer was reported to be due to up-regulation of the growth factor IGF-I [59]. Other non-cellular TME components—apart from soluble molecules and growth factors—include acidic environment (relatively low pH), hypoxic conditions, augmented reactive oxygen species (ROS) levels due to hypoxic conditions, extracellular matrix (ECM), etc. All these cellular and non-cellular TME components are essential for the survival and growth of the tumor and promotes angiogenesis, metastasis, tumor invasiveness, and increase in MDR proteins, thus contributing towards drug resistance development and reduced chemotherapeutics efficacy [60], therefore, in order to improve the efficacy of chemotherapeutics and reducing the drug resistance-appropriate targeting of cellular and non-cellular components of TME for rectification.

Another important factor that leads to cancer drug resistance is the high degree of tumor heterogeneity. Tumor cells heterogeneity is depicted by the presence of different cellular morphology, phenotypes, gene expression, epigenetics, metabolic, and transcriptomic features which are distinct from normal cells [61]. Tumor heterogeneity may be of two types: intertumoral and intratumoral. Intertumoral heterogeneity refers to the heterogeneity which occurs between different tumor patients with the same histology, but differences in somatic mutation, genetic variation, and environmental factors. In contrast, the intratumoral heterogeneity which occurs within the tumor contributes majorly to metastasis, drug resistance, and subsequent therapeutic failures [61]. The intratumoral heterogeneity may be derived from either heritable or non-heritable sources. The non-heritable sources of intratumoral heterogeneity include CSCs and phenotypes plasticity. CSCs represents the resistant minor population of cells which was originally present in the tumor population that promotes cancer initiation and progression and plays a significant role in cancer resistance development [62]. CSCs exhibit the abilities of self-renewal, cancer-initiation, differentiation, and metastasis due to their various unique features including overexpression of ATP-binding cassette (ABC) transporter proteins, anti-apoptotic proteins, DNA damage repair activity, aldehyde dehydrogenase (ALDH) activity, and activation of key pro-survival signaling molecules such as Notch and NF-kB. These properties enhance the mediated cancer drug resistance development of CSCs. In this regard, CSC-targeted therapy is expected to be a core for the development of effective anticancer therapeutics. This CSCs targeting-based strategy using multifunctional noncomplex to overcome drug resistance represents a promising novel therapeutic approach for the treatment of resistant cancer.

The other drug resistance mechanism might occur due to the inactivation of the drug or lack of activation. Inactivation of the drug involves complex mechanisms due to changes in enzymatic conditions during cancer disease progression. As a result of these enzymatic changes, drugs and other proteins interact with each other—or partially degrade or modify—which leads to drug inactivation, and thus, drug resistance [63]. The inactivation of drug and cancer drug resistance is quite common in some drugs such as platinum-based drugs, 5-flouro uracil (5-FU), methotrexate (MTX), tomudex, and irinotecan. Platinum-based drugs inactivation occurs through thiol glutathione (GSH) mediated by enzymes γ-glutamylcysteine synthetase and γ-glutamyl transferase, which synthesize GSH [49]. The inactivation of irinotecan is mediated by cytochrome P450 enzymes such as UGT1A1, which is highly expressed in the liver and colon [64]. Some metabolites, such as 5-flouro uracil (5-FU), methotrexate (MTX), and tomudex, do not activate in vivo and provide anticancer effects due to the absence of specific cellular activity [41,65]. Whenever these metabolites are active, they show cytotoxic effects and lead to cancer cell death. Taken together, the activation and inactivation of the drugs are mediated by different sets of enzymes.

The other significant factors responsible for the development of cancer drug resistance include the increased activity of the drug efflux pump inside the tumor cells. The overexpression of ATP-binding cassette (ABC) transporters, especially P-glycoprotein (P-gp), distinguishes between different chemotherapeutic agents and their increased efflux activity is consistently reported as one of the major causes of multi-drug resistance development in both in vitro and in vivo conditions [66,67]. Despite various strategies being utilized for overcoming drug resistance arising from different extrinsic and intrinsic factors, many of these anti-drug resistance approaches were unsuccessful in their clinical trials due to either unpredicted adverse effects or further genetic mutations [35,64]. As a result, clinical trials are ongoing to explore innovative techniques for combating drug resistance development without producing side effects.

## 3. Nanotherapeutics in Cancer Therapy

Chemotherapy serves as one of the most common treatment modalities for cancer. However, chemotherapeutic treatments are also associated with untoward toxicity to healthy tissues, due to the non-specific targeting and accumulation of drugs in the body. Other reasons for the failure of chemotherapy include the inadequate solubility of hydrophobic drugs, poor oral bioavailability, high toxicity, difficulty in penetrating biological barriers such as the blood–brain barrier, transport limitations, and low therapeutic index. [68,69]. The low bioavailability of drugs and hydrophobic nature of most drugs results in insufficient drug accumulation in tumors and limits their therapeutic outcomes. Conventional chemotherapeutic treatment faces the most difficult issue of MDR in patients receiving chemotherapy at the start of or following a treatment period [12]. The reasons for drug resistance include tumor heterogeneity, tumor microenvironment, drug inactivation, overexpression of drug efflux pumps, bypass of apoptosis process, genomic instability, epithelial to mesenchymal transition, and modulation of signaling pathways. Cancer drug resistance greatly impacts patients’ quality of life and poses a huge healthcare burden in terms of increased hospitalization and high costs related to healthcare. In order to overcome the several limitations faced by the conventional chemotherapeutic approaches, in last few decades, rapidly emerging nanotherapeutics-based strategies were explored for cancer therapy.

The nanotechnology-based approach involves the creation and manipulation of nanoscale size materials (1–1000 nm) that can interact with cell membrane and biomolecules present inside cells [70]. Several forms of nanomaterials (most commonly nanoparticles) were extensively utilized for targeted drug delivery for various diseases, including cancer. The cancer nanotherapeutics-based approach offers various advantages over conventional chemotherapy such as improved drug stability and solubility, prolonged half-lives of drugs in blood plasma, specific targeting of tumors with improved absorption, enhanced concentration of drugs at target site, ability to encapsulate a range of drugs, therapeutic payloads into the blood stream via targeted drug delivery with minimum systemic toxicities [71]. This nanotherapeutics-based approach for cancer therapy would improve the current cancer treatment potential, along with the management of drug resistance induced by CSCs. Nanoparticles-based platforms allow both the passive and active targeting of tumors. Solid tumors are generally hypervascular due to the upregulation of proangiogenic signaling pathways. However, the newly formed vessels indicate an abnormal architecture with the hyperpermeability tumor cells. The tumor mass also demonstrates poor lymphatic drainage, which allows the accumulation of macromolecules of a size of >40 kDa within the tumor microenvironment [72,73]. Thus, defective tumor vessels and impaired lymphatics in the tumor tissue allow the preferential accumulation of nanoparticles (NPs) in tumor vasculature and interstitial space by enhancing the permeability of the abnormal tumor microvasculature while suppressing the lymphatic drainage. The EPR effect is a fundamental prerequisite for nanoparticles-based targeted delivery to tumors. In order to benefit from the EPR effect, the optimal particle size should be in the range of 10 to 200 nm. If particles are too small, they will be cleared through the kidney and will not accumulate into the tumor site. In contrast, particles that are too large would not be able to penetrate tumor vasculature and interstitial space [74]. There is a substantial variation in EPR between patients and tumor types and sometimes even within the same patients or tumor type with time. Various researchers have demonstrated the stratification of cancer patients’ subpopulations based on the nanoparticle’s accumulation though EPR during preliminary clinical studies [75,76]. These reports indicate that EPR is a predictive marker and may have vital role cancer nanotherapies-related clinical success.

**Figure 4:**
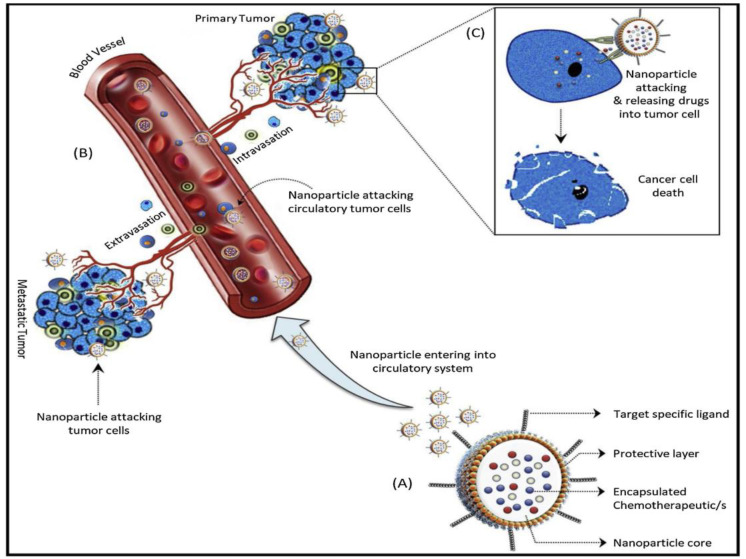
Cancer nanotherapeutics steps: (**A**) Nanoparticles with a protective layer loaded with chemotherapeutics of interest and decorated with target specific ligand. The drug loaded in the core of the nanoparticle can specifically recognize target cells using target-specific ligands. (**B**) Nanotherapeutics can reach primary and secondary tumors after entering the circulatory system and target specific tumor cells through the intravasation and extravasation processes. (**C**) Encapsulated chemotherapeutics are released after binding of tumor specific ligand and target cells surface receptors that causes cellular death. Reproduced from Ref. [34], (2022), with permission from Elsevier.

Cancer nanotherapeutics were extensively employed for the targeted delivery of drugs to tumors using different nanoformulations [77,78] (Figure 4). Despite their success and efforts to develop various non-invasive administration routes (oral, nasal, and transdermal) for nanoparticles, most cancer nanotherapeutics utilize the intravenous delivery route for systemic delivery to tumors [79]. In order to increase the effectiveness of chemotherapy, radiotherapy, and other cancer treatments such as CSCs targeting, several preclinical studies demonstrated the utilization of nanotherapies. After the last few decades, various nanoparticulate systems were developed and their drug delivery capacity was explored, aiming to overcome multi-drug resistance via targeting CSCs, overcoming efflux pumps, reducing some CSCs biomarkers, and inhibiting tumor growth. Moreover, nanoparticles-based therapeutics indicated promising results in terms of their low toxicity and biocompatibility; however, there are still concerns regarding their in vivo usage. In different strategies such as molecular targeting, magnetic hyperthermia, and photothermal and photodynamic therapy, the combination of metallic or polymeric nanoparticles and immunological approach was demonstrated successfully in specific tumor targeting.

Currently, numerous unique nanomedicines or structurally varied nanoformulations such as polymer conjugates, liposomal nanoparticles, polymeric nanoparticles, polymer micelles, organic nanoparticles, inorganic nanoparticles, metal nanoparticles, magnetic nanoparticles, nanogels, nanocrystals, dendrimers, carbon nanotubes, and hybrid nanoparticles, are being developed and employed extensively to reverse cancer drug resistance in various pre-clinical studies. Among these nanocarriers, liposomes, polymeric micelles, and polymeric nanoparticles have already reached clinical trials and also received FDA approval [12,34,69]. This can be seen in doxil, the first anticancer nanomedicine approved for clinical trials. Doxil is a liposome encapsulated doxorubicin which demonstrated an improved half-life compared to doxorubicin and with reduced cardiotoxicity [80]. Similarly, Abraxane, comprising of paclitaxel encapsulated within the albumin nanoparticles, indicated improved paclitaxel water solubility and a 28% reduction in death risk in metastatic pancreatic cancer patients when employed in a combination therapy with gemcitabine during phase III clinical trials [81]. Cancer nanotherapies using folate and transferrin receptor mediated nanotherapeutics also allow targeted delivery to tumor cells with significantly reduced damage to nearby cells which were otherwise damaged due to nontargeted conventional chemotherapy [82]. The different nanostructures employed in cancer therapeutics set forth therapeutic and/or theranostic properties with the ability to accommodate small/biomacromolecular therapeutic agents, contrast/imaging agents, and other therapeutic agents for therapy, as well as diagnosis. Moreover, the development of multimodal combination therapy utilizing multifunctional nanoparticulate systems to co-deliver combinations of different therapeutic cargos represents an attractive treatment option to surmount MDR.

In an earlier report, Wang et al. demonstrated an effective targeting and eradication of cervical CSCs [83]. In another report, sialic acid modified chitosan and poly (lactic-co-glycolic acid)-based nanoparticles loaded with curcumin inhibited the proliferation of glioblastoma cells and brain CSCs through targeting using the antibody against aldehyde dehydrogenase [84]. Several other reports also utilized similar strategies using different nanomaterials using co-delivery of drugs and indicated promising results in targeting hepatocellular carcinoma and liver CSCs [85,86], breast cancer tumor cells [87], ovarian CSCs [88], osteosarcoma cells [89], gastric CSCs [90], laryngeal stem cells [91], and glioma stem cells [92,93]. Furthermore, various nanotherapeutics-based approaches were employed to improve the treatment of glioblastoma as the prognosis is very poor with this cancer. In a preclinical study, poly (β-L-malic acid)-based nanobioconjugate was developed in order to block the expression of laminin-411 in glioblastoma cancer, which is reported to correlate with high tumor grade and overexpression of CD133 and notch signaling pathway (putative markers of CSCs) [94]. The developed nanoconjugate had the capability to cross the blood–brain barrier without showing any toxicity and indicated a significant survival of glioblastoma mice by inhibiting CSCs markers and the modulation of the notch signaling pathway. In a different approach, nanoparticles-based strategies were directed to target mitochondrial metabolism [95]. In a recent report, chitosan-gold nanoparticles were evaluated for their action on the acute T lymphoid leukemia cell line and chronic myeloid leukemia cell line. Herein, nanoparticles induced ROS production in both cell lines leading to mitochondrial impairment by loss of mitochondrial membrane potential without causing any detrimental effect on healthy immune cells [96]. Furthermore, enhanced sensitivity to some chemotherapeutics was observed using gold nanoparticles that inhibited cell proliferation and metastasis [97]. Nanoparticles were shown to have the potential to be a delivery vehicle for different anticancer therapeutic agents beyond their usual role as carriers for chemotherapeutics. The anticancer therapeutic agents incorporated in nanomaterials for cancer therapy include antisense oligonucleotides [98], DNA inhibitor oligonucleotides [99], small interfering RNA (siRNA) [100,101], molecularly targeted agents [102], and mRNA [103]. Furthermore, exosomes were also employed for anticancer payloads and targeting of tumors, owing to their endogenous origin [104]. In the past few years, nanotherapeutics-based strategies have already demonstrated in-depth innovation in cancer therapy. In this context, single nanoformulations integrated with both therapeutic and diagnostic functions present a promising approach for studying the disease progression, therapeutics accumulation and monitoring of pharmacokinetics in preclinical and clinical studies [105,106,107]. Multifunctional nanoparticles provide insight into the tumor heterogeneity within patients and allows for the development of potential personalized patient specific therapy [108]. Several therapeutic nanoparticle (NP) platforms, such as liposomes, polymeric micelles, and albumin nanoparticles are FDA-approved for cancer treatment. Numerous nanotechnology-enabled therapeutic modalities are being investigated in clinical trials, including improved chemotherapy, radiation treatment, photo thermal therapy (PTT), photodynamic therapy (PDT), magnetic hyperthermia, RNA interference (RNAi) therapy, and immunotherapy [37]. Currently, nanomedicines have taken superiority as a treatment option for overcoming cancer drug resistance. However, due to the rapid development and widespread usage of nanomaterials, limited evaluation of their safety and efficacy data related to nanomedicines are available regarding clinical applications. This further substantiates the need for high quality clinical trials for better understanding their use and safety. Taken together, cancer nanotherapy presents an attractive alternative strategy in combination with other treatments to conventional chemotherapy, particularly that against CSCs. Despite its success and the approval of a few other nanotherapeutics approaches for cancer therapy, still there is long way to go before these reach clinics and more studies are required to manifest their safety for human use in cancer treatment.

## 4. Emerging and Innovative Nanotherapeutics-Based Strategies against Drug-Resistant Cancers

Nanotherapeutics serve not so much to overcome the chemotherapeutic treatment, but rather to overcome the chemoresistance of cancers, improve pharmacokinetics of the drugs, and decrease or eliminate their systemic toxicity, etc. The foremost objective of the nanotherapeutics-based approach is to target specific cancer cells and their microenvironment with minimal toxicity by delivering chemotherapeutic agents efficiently to the target site. Moreover, the development of nanotherapeutics in the past few years indicates its considerable potential in the cancer therapeutic domain. Aside from cancer therapies, nanotechnology-based medicines have significant potential implications in the diagnostic imaging of many drug-resistant cancers. Nanoscale delivery systems for cancer-specific targeting have demonstrated enormous potential in the past few years with the development of strategies for specifically targeting specific cells, particularly CSCs, the tumor microenvironment, and various tumor components, using a variety of emerging and innovative approaches. The innovative approaches include the nano-therapies based approach to target specific components of the tumor environment (cellular and non-cellular component), employment of RNA interference technique (siRNA and miRNA based specific delivery), self-assembly based prodrug-based approach, exosome-based delivery, stimuli responsive delivery, advanced delivery systems for targeting the CSCs and integrin, and others for specific cancer therapy [17,34,43]. In this section, various emerging and innovative strategies currently ongoing for specific targeting of tumor cells and microenvironment were reviewed and discussed in detail along with their advantages and associated challenges.

### 4.1. Nanotherapeutis-Based Approaches for Targeting Tumor Microenvironment (TME)

Tumor microenvironment (TME) plays a vital role in imparting tumor heterogeneity and disease progression. The heterogeneity of TME and its components, such as cells, interstitial fluid, and ECM, act as physical barriers and do not allow drugs to permeate the tumor tissue. As a result, there are marked gradients of cell proliferation and drug concentrations which influence the tumor sensitivity towards drug treatment [109]. This condition induces anticancer drug resistance. MDR presents major unresolved challenges in cancer chemotherapeutics and about 50% of patients face tumor relapse problems due to MDR. TME and its components induce drug resistance through a variety of processes, including cell–cell and cell–ECM interactions, crosstalk between distinct cells, phenotypic changes, mechanosensing variation, and protective dormancy. Furthermore, additional factors—including the overexpression of efflux pumps such as ATP-binding cassette (ABC) transporters and P-glycoprotein (P-gp)—found on certain cancer cells contribute to drug efflux and resistance [110]. TME allows tumor cells to avoid the harm produced by traditional clinical cancer therapies such as chemotherapy, radiation, and surgery.

**Figure 5:**
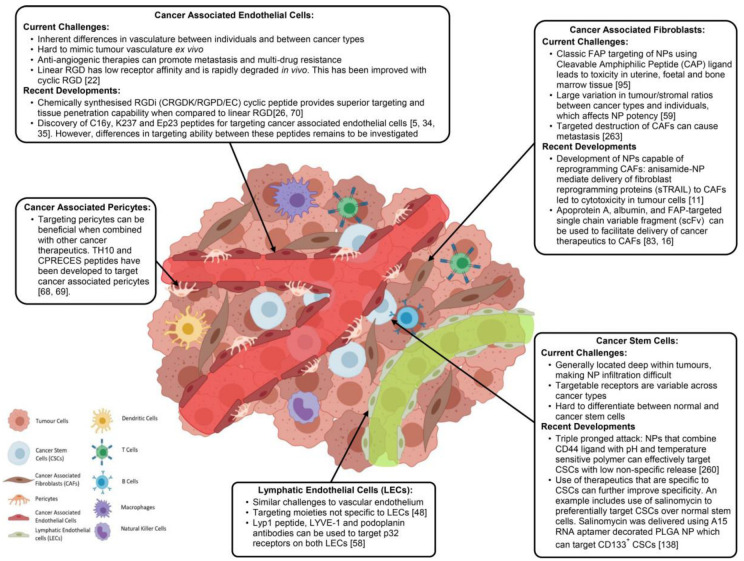
Cellular components of tumor environment targeted by nanoparticulate system for cancer therapy. Reproduced from Ref. [115], (2022), with permission from Elsevier.

The local microenvironments of tumor cells and crosstalk between specific cancer cells plays a crucial role in tumor progression was elucidated more than a century ago according to Stephen Paget’s seed and soil hypothesis [111]. However, the role of non-neoplastic cells of TME in tumor development and metastasis was uncovered only in the last three decades [112]. TME comprises both cellular and non-cellular components that play critical roles in the development of drug resistance. The cellular component of TME includes cancer associated fibroblasts, cancer associated vascular endothelial cells, cancer associated pericytes, cancer associated immune cells, lymphatic endothelial cells (LECs), and CSCs. The cellular components of TME by different nano-drugs systems are highlighted in Figure 5. Non-cellular component characteristics of TME include hypoxia, an acidic environment, the extracellular matrix, cytokines, growth factors, and vascular networks [113]. TME’s non-cellular components create a favorable and permissive environment for cancer cell proliferation. TME exhibit characteristics that separate them from normal tissue include their leaky vasculature, inadequate vascular perfusion, an acidic environment, changed pH dynamics, altered enzyme expression, altered metabolism, and hypoxic circumstances [114]. All these regions provide therapeutic opportunities which are exploited by nanocarrier-based drug delivery systems. In order to design chemotherapeutic and chemo preventive strategies to overcome drug-resistant cancers, in-depth knowledge of tumor biology is pertinent. Consequently, the targeting of both cancer cells and tumor microenvironment is necessary to achieve superior therapeutic efficacy. Therefore, in order to develop improved and efficient drug delivery systems, TME modification is a prerequisite through the better understanding of both TME stromal components functioning and its morphological features [24].

**Figure 6:**
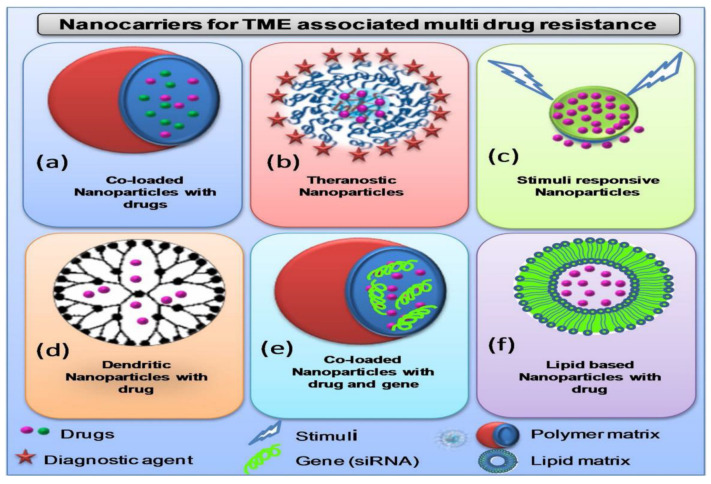
Different nanocarriers being utilized for targeting TME to overcome multi-drug resistance: (**a**) Nanoparticles loaded with two different drugs for co-delivery at target site for synergistic therapeutic action. (**b**) Theranostic nanoparticles co-loaded with both therapeutic and diagnostic agents. (**c**) Stimuli responsive nanoparticles respond against different components of TME such as pH change, ions change, different oxygenation. (**d**) Multifunctional branched polymeric dendrimer-based nanocarrier loaded with drugs. (**e**) Nanoparticles loaded with both drugs and nucleic acids (siRNA, miRNA) for synergistic therapeutic action (**f**) Liposomes loaded drugs for targeted delivery. Reproduced from Ref. [24], (2022), with permission from Elsevier.

In the last two decades, various other cellular components of TME, such as CSCs, endothelial cells, and stromal cells, were identified and their role in tumor growth is established. All these cellular components vary greatly in terms of size, morphology, and expression of surface receptors, paving the way for us to target these cells individually in order to produce synergistic therapeutic effects [115]. The advancement of TME-enabled nanotherapy in the past few years demonstrated promising strategies and approaches for the modulation and targeting of TME in combating drug-resistant cancers by limiting disease progression [116]. Furthermore, a number of novel smart nanoparticles with transformational properties exhibited improved spatiotemporal control over particular tumor microenvironmental targeting. Because of their customizable size, surface coating, and capacity to include a vast number of therapeutic drugs, nanoparticles (NPs) have emerged as a viable platform for TME targeting. Emerging nanocarriers are being utilized for targeting TME and its components include nanoparticles (polymer- and lipid-based), liposomes, polymeric micelles, magnetic nanoparticles, polymer drug and nanoconjugates. A variety of nanocarriers are employed for targeting TME in order to overcome multi-drug resistance (Figure 6).

**Figure 7:**
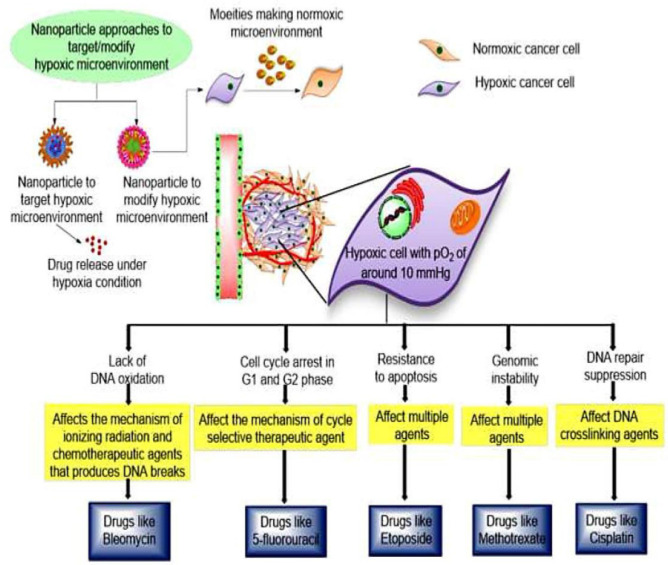
Cancer nanotherapeutics approaches to counter hypoxic conditions within tumor microenvironment, which is prime contributing factor for drug resistance. In this approach, specific drugs that can counter hypoxic environment are loaded within nanocarriers. Nanoparticulate system further specifically releases drugs in tumor microenvironment to modulate the hypoxic environment and causes cell death. Reproduces from Ref. [119], (2022), with permission from Elsevier.

TME modulation and targeting using nanocarriers can be achieved either through passive targeting or active targeting. In the passive approach, tumor targeting is carried out by diffusion process and EPR effect is considered as crucial factor. In passive targeting, accumulation of nanocarriers is supported by abnormal leaky vasculature of tumor compartment. Nanocarriers are generally functionalized with specific ligands such as folic acid, transferrin, and aptamers in active targeting that could interact with overexpressed folate and transferrin specific receptors present on targeted cells. In active targeting, different ligands not only target cellular components of TME, but also non-cellular components such as hypoxic conditions and acidic environment. Physiological hypoxic conditions in tumor microenvironment contributes primarily to the tumor growth and cancer drug resistance (Figure 7).

In cancer nanotherapeutics targeting TME, monotherapy sometimes fail to produce the desired therapeutic effect. Thus, other strategies such as multifunctional nanomedicine and combination therapy were employed for enhancing the effectiveness of cancer therapy. Multifunctional nanomedicine utilizes encapsulation of various therapeutic cargos such as chemotherapeutic drugs, clustered regularly interspaced short palindromic repeats (CRISPR) nucleotides or RNA interference (RNAi) [117,118]. Thereafter, nanoparticles can migrate to target tumor sites to release therapeutic agents in a controlled manner through local or systemic administration.

Various nanoparticle-mediated approaches were reported to target TME in the past few years by creating nutrient deprived conditions for cancer cells together with exposure to various destructive mechanisms. Nanotherapeutics-based strategies are not only limited to improving chemotherapy, but also incorporate gene therapy and its applications for diagnostic and theragnostic domain. Consequently, nanoparticle-based approaches are reported to utilize either of the two mechanisms and expanded the nanotherapeutics in both directions. Currently, there are only a few clinically approved nano pharmaceuticals available in the market to treat cancer, namely, doxil^®^, Abraxane^®^, and Genexol^®^. A list of the nanoparticles-based approaches for targeting cellular and non-cellular components of TME is presented in Table 2 and Table 3.

Nanotechnology-based products have shown promising outcomes in targeting TME and a few products are now clinically approved; however, their applications remain limited in treating certain types of cancers (although not all) [169]. TME provides conflicting attributes because, on the, it allows improved nanoparticles accumulation due to its leaky vasculature, and on the other hand, it also acts as a barrier for nanoparticles extravasation [161]. The TME barriers’ contributions toward nanoparticles extravasation include high interstitial fluid pressure (HIFP), pericytes coverage, basement membrane, and composition of ECM. The interstitial fluid of the tumor environment is similar to blood plasma and comprises 50–60% of plasma proteins and electrolytes composition; however, the interstitial fluid pressure varies. The tumor IFP is elevated (5–40 mm Hg) compared to normal IFP (range of −3 to 3 mm Hg). The pressure increases as the tumor growth progresses due to various factors such as rapid cell proliferation, presence of highly crosslinked collagen, modulated extracellular matrix, increased contractions of stromal cells, lack of pericyte coverage, high vascular permeability, lack of lymphatic drainage, and increased secretion of angiogenic factors and growth factors [24]. High tumor IFP exerts mechanical forces on cells and stimulates the proliferation tumor cell proliferation [170]. Pericytes coverage presents another barrier for nanoparticles-mediated drug delivery. An earlier report indicated that pericytes dysfunction leads to loss of vascular coverage and plays an important role in disease progression [171]. Basement membrane represents another barrier of TME which performs the function of a sieve to modulate the nanoparticle extravasation from blood capillaries to the TME. Although the basement membrane does not induce the elevation of IFP, its structural complexity and thickness restricts the entry of nanoparticles or therapeutic agents’ migration to TME [169]. Furthermore, ECM composition, and structural and componential complexity restrict the extravasation of nanoparticles. Apart from the ECM composition, nanoparticle distribution is influenced by the alignment and orientation of collagen fiber network. In addition to tumor cell growth, stromal cell density contributes to the solid stress by compressing the matrix into a disordered network and restricting nanoparticle penetration, both of which limit nanoparticle penetration. A general decrease in nanoparticles that extravasates from neighboring micro vessels compromises the ability of stromal cells to internalize therapeutic NP in cancer cells. Taken together, TME barriers such as the presence of stromal cells coverage, extensively cross-linked collagen networks, and interstitial fluid pressure, among others, restrict the entry of chemotherapeutic agents from reaching the target cell. This restriction ultimately reduces the therapeutic benefits in patients. Therefore, the remodeling of cellular and non-cellular components of TME is pertinent in order to improve drug delivery by facilitating the extravasation of nanoparticles to TME. The four main strategies employed for the enhancement of nanoparticles extravasation include the vascular normalization strategy, stress alleviation strategy, and stromal/tumor matrix normalization strategy [24,172]. The normalization of the vascular system, mediated, for example, by the metronomic dosage of some conventional chemotherapy (such as docetaxel), may enhance blood flow inside the tumor, but it also closes the pores in the capillary walls, which are typical of solid tumors and required for the EPR effect. As a result, vascular normalization may even limit the growth of nanosized systems within malignancies. All three strategies employ different nanoformulations such as nanoparticles and polymer micelles to enhance extravasation. The priming mechanisms of stromal normalization strategies include the degradation of ECM, modification of ECM, reduction of collagen content, and reduction of IFP. In the context of the vascular normalization strategy, blocking of VEGF receptors, vessel diameter reduction, inhibition of tubulin, and stromal cells present main priming mechanisms. Furthermore, the prime mechanisms of the stress alleviation strategy involve the inhibition of tubulin, depletion of stromal cells, and reduction of IFP. In the past few years, TME-responsive cancer nanotherapeutics showed fast development, with the design and development of various theranostic strategies for combating drug-resistant cancers. Although few nanoparticles formulations are currently in clinical trials, the multitude of pre-clinical testing being far more than for clinical testing presents an obvious barrier for translation into clinical settings. Although TME-enabled nanotherapy showed high performance outcomes for further clinical translation, still a number of challenges must be overcome to ensure the better feasibility of these targeted systems [37]. In order to enhance the clinical translatability of nanoparticles platforms, safety profile, formulation scalability, targeting efficiency, and selection of pre-clinical models act as major determining factors.

### 4.2. Nanotherapeutic Strategies for Targeting Cancer Stem Cells (CSCs)

Tumor heterogeneity represents major obstacle in cancer therapy as bulk of tumor harbors various cell types with differential sensitivity to chemotherapy [61]. One of the crucial factors responsible for tumor heterogeneity is considered as CSCs, which regulates the tumor microenvironment and exhibits self-renewal ability, invasiveness and high tumorigenicity [173,174]. CSCs are small group of cancerous cells responsible for tumor initiation, progression, relapse, and poor prognosis, highly influencing the available therapeutic processes; see [175]. CSCs are able to resist conventional therapies such as chemotherapy and radiotherapy owing to their intrinsic characteristics such as phenotypic plasticity capacity, maintenance of a slow dividing state, drug efflux transporters, overexpression of antiapoptotic proteins, highly efficient DNA repair system, detoxifying enzymes epithelial to mesenchymal transition, and sustained stemness features [176,177,178]. Additionally, CSCs’ persistence in a hypoxic tumor microenvironment confers additional resistance to anticancer therapy [17]. Moreover, CSCs represents an important source responsible for resistance to traditional chemo and radiotherapy. Therefore, the development of efficient anticancer strategies which would specifically kill both tumor cells and CSCs would form the core of cancer therapeutics.

In the past few decades, several researchers studied CSCs properties and emphasized finding different ways to specially targeting the CSCs population for improving conventional chemotherapeutic approaches [14,15,179]. In order to attempt this, nanotherapeutic-based approaches using nanoparticles were developed for specific targeting of CSCs to reduce the chances of cancer recurrence and provide better palliative care. The potential nanotherapeutic approaches utilized for targeting CSCs in the past few years include crucial factors required for the survival of CSCs in the tumor microenvironment, such as specific surface biomarkers (CD44, CD133, EpCAM, aldehyde dehydrogenases), drug efflux pumps (ABC transporters) expression, different metabolic pathways, and signaling pathways (Wnt/β-catenin, Notch and Hedgehog) [178,179].

Recent research efforts in understanding the properties and different mechanisms of targeting CSCs paved way for the development of innovative nanotherapeutics for targeting CSCs. One of the most important overexpressed markers on the surface of CSCs is the cluster of differentiation-44 (CD44). Rao et al. developed polymer nanoparticles with chitosan coating and loaded with chemotherapy agent for targeting CD44. The results demonstrated increased therapeutic efficiency in mammary tumor spheroids model as nanoparticles delivered to tumor microenvironment specifically targeted CD44 overexpressing CSCs due to the high affinity between CD44 receptors and chitosan [180]. Furthermore, CSCs targeted nanotherapeutics gained much attention and other important biomarker CD133 was also utilized for specifically targeting CSCs. However, a pertinent issue related with the utilization of CD44 and CD133 lies in selective removal of a subset of CSCs only and may promote phenotypic shift and differentiation in tumor unintentionally. This leads to the compensatory high proliferation of cells and ultimately promotes chemotherapeutic resistance [181]. Therefore, the utilization of a more ubiquitous marker that can better target the large population of CSCs would be a more feasible approach. Thereafter, researchers utilized riboflavin loaded intracellular vesicles with coating of ATP binding cassette subfamily G member 2 (ABCG2) for targeting CSCs specifically and they observed a higher accumulation of riboflavin within cytoplasm due to specific recognition properties [182]. In another study, a pH responsive/hypoxia responsive riboflavin linked three-pronged nanoparticles were utilized for targeting both tumor cells and CSCs [183]. Herein, nanoparticles are loaded with three drugs, namely, irinotecan, cyclopamine, and erlotinib, which are able to kill undifferentiated CSCs, differentiated breast cancer specific MCF-7 cells and vascular niches in tumor microenvironment, respectively. Similarly, irinotecan conjugated riboflavin displayed exceptional anticancer efficacy with increased accumulation inside cancer cells. Wang et al. moved on to use salinomycin-loaded nanoparticles to selectively target and kill cervical CSCs [83]. In another study, chitosan poly (lactic-co-glycolic acid)-based nanoparticles loaded with curcumin and modified with sialic acid demonstrated blood–brain barrier permeability and inhibited proliferation of glioblastoma cells and brain CSCs through targeting the aldehyde dehydrogenase of CSCs [84]. In a recent nanotherapeutic strategy, nanoparticles co-loaded with chemotherapeutic drug, camptothecin, and differentiation-inducing agent, all-trans retinoic acid, demonstrated CSCs killing within tumors via dual strategy. The dual strategy involves first the promotion of CSCs differentiation in hypoxic conditions that lead to increase of reactive oxygen species; second, the promotion of the release of camptothecin and subsequent death due to increased levels of reactive oxygen species. This strategy reduces stemness0related drug resistance, enhancement of the chemotherapeutic and prevention of post-surgical tumor relapse response with controlled drug release in breast cancer models [184].

With the recent advancements in cancer nanotherapeutics, various emerging and innovative strategies have shown immense potential in targeting CSCs using photothermal therapy, magnetic hyperthermia, photodynamic therapy, and molecular targeting. The photo thermal therapy (PTT) field has shown promising results for the CSCs targeting nanotherapeutic approach as this method stimulates hyperthermic physiological responses with the conversion of light into heat using metal nanoparticles to eradicate CSCs [71]. Tian et al. utilized gold nanospheres functionalized as a surface biomarker for osteosarcoma stem cells, CD271 for targeted PTT, and reported the inhibition of cells and targeted death in osteosarcoma treatment [185]. Another promising strategy utilized a biocompatible polymeric micelles-based nanocarrier co-loaded with gold nanorods and Adriamycin for killing CSCs under laser ablation via targeting an important CSCs surface marker, EpCAM [186]. In another study, a nanoparticle system based on electrospun polycaprolactone nanofibers encapsulating all-trans retinoic acid and hydroxylated multi-walled carbon nanotubes for targeting and killing glioma stem cells was presented. Herein, stem cells inhibition was displayed by increasing the local temperature under near-infrared illumination, which further suggests its increased sensitivity towards heat treatment [187]. In another strategy used to overcome the resistance of CSCs, Wu et al. employed nanoparticles coated with the membrane of melanoma cells for simultaneously targeting chemotherapy, photothermal therapy, and photoacoustic imaging. The results reported this strategy’s enhanced targeting ability, along with excellent tumor ablation rate, and antitumor efficiency [188]. Another potential light-triggered minimal invasive cancer therapy for targeting CSCs includes photodynamic therapy (PDT) [189]. PDT produces reactive oxygen species (ROS) and free radicals with activation of a specific wavelength of excitation light and related to photosensitive agents in tumor tissues. PTT-based treatment promotes the autophagy, apoptosis, and necrosis of tumor cells, suggesting its role in reversing chemoresistance [190]. Crous et al. employed nanobioconjugate along with the photodynamic effects and indicated the significant destruction and eradication of lung CSCs [191]. In another study, nanoparticles loaded with a bimodal metallacage and with PDT targeted CSCs by decreasing the cells mobility under laser irradiation [192]. In a similar approach, a combination chemotherapy wherein nanoparticle-based micelles were loaded with photosensitizer (mitoxantrone) and anti-EpCAM–CSCs biomarker reported better antitumor efficacy compared to either near infrared irradiation or chemotherapy alone with simultaneous chemotherapy and PDT [193]. Cao et al. utilized MnO_2_@Ce_6_ nanoparticles and a PDT-based approach which revealed improvement in tumor microenvironment related therapy resistance by modulating tumor microenvironment by excess hydrogen protons and H_2_O that resulted in subsequent eradiation of CSCs [194]. Furthermore, the nanotherapeutics approach combining both PTT and PDT was utilized and showed a beneficial role in minimizing the metastasis of different cancer types by specific CSCs targeting. Another nanotherapeutic approach for targeting CSCs includes magnetic hyperthermia using magnetic nanoparticles wherein increased cancerous tissue temperature serves as an operative therapy for cancer therapeutics [195]. Magnetic nanoparticles are used for cancer therapy in this technique because of their beneficial physiochemical qualities, such as size resemblance to biomolecules, magnetic properties, appropriate combination capabilities, and targeted drug delivery capacity [196]. Su et al. utilized superparamagnetic iron oxide nanoparticles modified with the anti-CD44 antibody and alternating magnetic field resulting in the significant inhibition of CSCs growth and subsequent death in the head and neck squamous cell carcinoma model via magnetic fluid hyperthermia [197]. In another study, a mesoporous silica nanoparticle under an alternating magnetic field demonstrated an efficient inhibition of tumor growth with the elimination of CSCs through the blockage of the hypoxia signaling pathway and hyperthermia [70].

Molecular targeting is another nanotherapeutic technique for targeting specific CSCs by changing molecular and metabolic processes. MicroRNA21 is an oncogenic gene that, when overexpressed in triple-negative breast cancer, downregulates several tumor suppressors. As a result, downregulation would improve tumor suppression and reverse resistance. To attempt this, Yin et al. employed a three-way junction motif with the utilization of nanoparticles conjugated with the inhibitor of microRNA21, RNA aptamer and CD133 receptor for CSCs targeting. This approach specifically targeted both the triple-negative breast CSCs and cancer cells and indicated reduced cancer cell migration and upregulated tumor suppressors’ expression in in vitro and in vivo studies [198]. Nanotherapeutics based on molecular targeting constitute a more effective way of targeting CSCs, resulting in tumor growth suppression and metastasis reduction via decreased CSC adhesion, migration, and number [199]. Taken together, nanotherapeutic techniques for targeting CSCs demonstrate enormous promise and may enable us to overcome cancer treatment resistance. However, further understanding and the study of novel target molecules and CSC characteristics will be necessary in the future to convert these techniques into clinical practice.

### 4.3. siRNA-Based Nanotherapeutic Strategies

Currently, targeting the suppression of the oncogenes’ expressions along with targeted chemotherapy shows tremendous success and represents one of the foremost strategies in cancer treatment. Earlier, different gene therapy-based approaches were utilized for their knockdown of genes associated with cancer pathophysiology; however, none of them were able to provide the complete suppression of genes [200]. Thereafter, an alternative innovative genetic approach RNA interference (RNAi) was developed for the inhibition of specific messenger RNA (mRNA) expression by controlling uncontrolled cell growth and proliferation, especially in carcinoma cells [201]. The RNAi approach triggers a homology-dependent degradation of targeted mRNA and reversible specific gene silencing capability through the delivery of non-coding double stranded RNA (dsRNA) to cancer cells [202]. In RNAi, the non-coding short double stranded RNAs include short interfering RNAs (siRNAs) and micro RNAs (miRNAs), which show broad potential as therapeutics by silencing sequence-specific genes. In this section, we discuss siRNA delivery-based strategies for cancer therapy; miRNA-based delivery is discussed in the subsequent section.

The basic strategy involved with siRNA delivery-based gene silencing involves the rational design of siRNA-based delivery systems and identification of targeted genes for the selective knockdown of susceptible oncogene expression. Free siRNA is anionic and hydrophilic dsRNA, with an average diameter of <10 nm, which prevents them from readily crossing cell membranes. The physicochemical and pharmacokinetics properties of siRNA such as short half-life, toxicity, reduced cellular uptake, and degradation vulnerability by serum nucleases, limit the in vivo systemic administration of naked siRNA. Nevertheless, naked siRNAs are rapidly cleared by cells through opsonization and phagocytosis processes by the mononuclear phagocytic system as a part of routine immune system-mediated clearance of foreign substances [203]. Furthermore, siRNA delivery into the targeted tissues is impeded by the presence of different biological barriers that ultimately hinder its effectiveness in vivo. Therefore, different delivery vehicles are required for transporting siRNA to the site of action in order to achieve the clinical potential.

With advancements in the domain of nanotechnology, nanoparticles with remarkable physicochemical features serve as the vehicle of choice for siRNA targeted delivery [25]. Nano-encapsulated siRNAs modifies its pharmacokinetic properties by improving the solubility, oral bioavailability, serum stability, and renal and hepatic elimination owing to their diminutive size. Moreover, encapsulating siRNA into nanoparticles improves cellular internalization and intracellular drug release while decreasing cancer cell resistance to siRNA employing stimuli-mediated nano-therapeutics [204]. Clinical application of siRNA-based nanotherapies siRNA-based nanotherapeutics for cancer therapy offers several advantages over chemotherapeutic anticancer drugs, especially the undruggable targets in cancer treatment. The first and foremost advantage is the high degree of safety. Second, siRNA acts at the post-translational stage of gene expression; therefore, there is no interaction with DNA. As a result, risks of mutation and teratogenic risks that are more common with conventional gene therapy are negligible. Third, siRNA is highly efficacious and preferentially target any genes with minimal off-target effects and immunogenicity [205]. Fourth, siRNA-based delivery systems can be easily fabricated and modified [206]. Fifth, siRNA therapeutics exhibit a promising antiproliferative and tumor growth suppression effect through different signaling pathways [207]. Sixth, they can cause angiogenesis suppression by inhibiting VEGFs and VEGFR-1 receptors [208]. Seventh, the inhibition of tumor invasion and metastasis is conducted through the utilization of different chemokines CXCL8 and CXCL11 [209]. Eight, unrestricted choice of specificity and targets compared to other antibody-based drugs or small molecule drugs are advantageous.

To date, there are several reports demonstrating its role in tumor treatment using nanoparticles-encapsulated siRNA-based delivery system. There are three main types of siRNA-based delivery systems in cancer chemotherapeutics, namely, lipid-based systems, polymers-based systems, and siRNA conjugates. In the lipid-based system, in order to form lipoplexes different cationic lipids, such as 1,2-dioleoyl-3-trimethylammonium propane (DOTAP), N-trimethylammonium chloride (DOTMA), and N-[1-(2,3-dioleoyloxy) Propyl]-N, N, were utilized along with neutral lip‘ids, such as cholesterol (Chol), 1,2-dioleoyl-sn-glycero-3-phosphoethanolamine (DSPE), dioleoyl phosphatidylethanolamine (DOPE), and 1,2-dioleyl-sn-glycero-3-phosphocholine (DOPC). In lipoplexes, the incorporation of siRNAs into positively charged liposomes is carried out by electrostatic interactions [210]. In the polymer-based siRNA delivery system polyethyleneimine (PEI), poly-L-lysine (PLL) chitosan, cyclodextrin, hyaluronic acid, and poly ethylene glycol (PEG)-based nanocarriers were extensively utilized [211]. In the siRNA conjugate system, antibodies, aptamers, peptides, and dendrimers were utilized. Among these abovementioned siRNA delivery systems, lipid-based delivery system attracted much attention in cancer therapy, and a few are already in clinical trials. A variety of domains such as cell proliferation and cell cycle progression, tumor microenvironment, angiogenesis, tumor invasion, metastasis, and chemotherapeutic resistance are targeted by siRNA-based nanotherapeutics in the preclinical studies listed in Table 4.

Since the last few decades, researchers and pharmaceutical industries focused on clinical studies using siRNA-based nanotherapies which were initiated in 2010 and several synthetic siRNA-based nanotherapeutics were explored in the past few years for treating recurrent and aggressive tumors. The first clinical trial of nanoparticles-mediated siRNA delivery CALAA-01 was published in 2010 by Calando Pharmaceuticals [100]. CALAA-01 comprises different components such as cyclodextrin-based polymer (CDP), external PEG chains to improve the stability of nanoparticles in biological fluids, a human transferrin protein (TF) to target TF receptors (TFR) on cancer cells surface, and a siRNA specific for M2 subunit target of the ribonucleotide reductase protein (RRM2). Moreover, intratumoral downregulation of RRM2 leads to the induction of apoptosis in cancer cells [230]. However, this study was only preliminary as it utilized only small set of patients. Thereafter, in 2014, the phase I clinical trial of liposomal siRNA-based delivery system termed as Atu027 was published by Silence Therapeutics GmbH. The structure of Atu027 contains a neutral, fusogenic DPhyPE helperlipid, PEGylated lipid MPEG-2000-DSPE (molar ratio: 50/49/1), and a AtuFect01 for targeting protein kinase N3 [101]. The phase I clinical trial dose-escalation of Atu027 demonstrated disease stabilization for 41% of patients suffering from metastatic pancreatic cancer. The efficacy of Atu027 was tested together with gemcitabine in a clinical trial for the treatment of cancer. Another clinical study employed using the biodegradable polymer matrix loaded with KRASG12D-targeting siRNA for prolonged delivery regionally within the tumor tissue by Silenseed Ltd. A phase I/IIa clinical study was conducted using this delivery system together with gemtabicine in patients with non-operable locally advanced pancreatic cancer. The results of clinical trial demonstrated no evidence of tumor progression and disease stability [231]. Furthermore, a multinational randomized phase II clinical trial using this delivery system is currently in progress. Another clinical study using a lipid nanoparticles-based siRNA delivery system called DCR-PHXC-101 was developed by Dicerna pharmaceuticals for downregulating the expression of the transcription factor Myc. In this dose-escalation clinical study, safety, pharmacodynamics, pharmacokinetics, and clinical activity of DCR-MYC were explored in patients with lymphoma I, advanced solid tumors, and multiple myeloma. Among all patients receiving treatment, the majority of patients demonstrated shrinkage in tumor and sustained metabolic response [232]. The most recent anticancer siRNA-mediated nanotherapeutics clinical trial conducted was using EphA2-siRNA-DOPC. Herein, EphA2, tyrosine kinases receptors serve as the target protein. The upregulation of EphA2 was reported in several studies related to breast, prostate, lung, pancreas, and most importantly, ovarian cancer, and causes tumor invasion, metastasis and angiogenesis. Herein, EphA2-siRNA was encapsulated in liposomal nanoparticles 1,2-dioleoyl-sn-glycero-3-phospahtidylcholine (DOPC) and combinedly termed as EPHARNA (EphA2-siRNA-DOPC) for their specifically target of EphA2 expression in the tumor [233]. The simultaneous administration of EPHARNA and paclitaxel demonstrated an anti-angiogenic effect and drastic reduction in tumor growth in several in vitro and in vivo studies [234]. Other in vivo toxicological studies reported no observed adverse events and no major toxicities at a dose range of 75–225 mcg/kg after a single or double administration of DOPC nanoliposomes [235]. The phase I clinical trial of EphA2-siRNA-DOPC started in 2015, with patients suffering from advanced metastatic solid cancer receiving two weekly intravenous doses over two hours of EPHARNA, and is still continuing [236]. A list of nanoparticles encapsulated siRNA engaged in clinical trials is enumerated in Table 5.

Although the lipid nanoparticles-mediated delivery of siRNA using ApoE coated lipid nanoparticles indicated high internalization into liver cancer cells, the siRNA-based delivery systems for other cancers are still under exploration. Despite the promising results of the improved siRNA delivery system for cancer treatment and several clinical trials, still not a single anticancer siRNA drug has been FDA approved for commercial usage [242]. This might be due to the problems associated with delivery to target tissues. As siRNA presents a huge potential for cancer treatment, in addition to the identification and utilization of internalization pathways for specific target cells, attempting to overcome the delivery problems would pave a way to the design of innovative siRNA-based delivery systems for cancer therapeutics.

### 4.4. MicroRNA (miRNA)-Based Nanotherapeutic Strategies

In the past few years, RNA-based therapeutics have shown immense potential in cancer nanotherapeutics. RNA-based therapeutics can be mediated either as inhibitors of target protein expression using siRNA and miRNA or as upregulators using mRNA [243]. miRNA-based cancer therapeutics have shown tremendous implications in the pathophysiological processes of cancer as emerging gene regulators. miRNAs are tiny, endogenous, noncoding RNAs that control gene expression in a variety of physiological activities, including cell growth and proliferation, differentiation, cell cycle, apoptosis, and tissue development [244]. The deregulated miRNAs affect the multiple biological pathways and leads to cellular transformation, malignancy, and cancer progression [245]. The differential expression of miRNAs in different tissues related to cancer enables them to target a multitude of transcripts related to cancer signaling pathways. The upregulation and downregulation of miRNAs leads to the suppression of tumor suppressor genes and increased expression of oncomers, respectively, which indicate their functions as both oncogenes and a tumor suppressor. For example, miR-10b, miR-125b, and miR-145 are downregulated, while miR-21 and miR-155 are upregulated in cancer development, suggesting their dual roles as tumor suppressors and oncogenes, respectively [246,247]. Owing to miRNAs’ functions as both tumor suppressor and oncogenic miRNAs, they can modulate multiple signaling cascades related to cancer and metastasis via the transcriptional effect. Therefore, miRNAs can be targeted in cancer therapeutics either as synthetic anti-miR sequences for an upregulated miRNAs or as miRNAs mimics for downregulated miRNAs [248]. In this context, miRNAs may be silenced to upregulate the tumor suppressor genes or degrade the anti-apoptotic genes. Taken together, the regulatory potential of miRNAs makes them a new, promising, individualized therapeutic strategy for cancer therapeutics.

In the past few decades, several miRNAs-based delivery systems were studied; however, their clinical translation was limited due to their short half-life, degradation by nucleases, very low endosomal and/or lysosomal degradation, broad functionality, and off-target effects. In order to overcome these problems, nanotechnology-integrated miRNA delivery systems were developed for the cell-specific delivery of therapeutic miRNAs/anti-miRNAs using targeted miRNA mimics. Several nanoparticles-based platforms, such as lipid-based nanostructures, polymer-based nanomaterials, inorganic nanomaterials, dendrimers, polymeric micelles, and bioinspired nano vehicles, were employed for miRNA delivery in the past few years for targeted delivery [243]. Earlier studies utilized inorganic silica-based nanoparticles as a vehicle for miRNA delivery and demonstrated the delivery of miR-34a to neuroblastoma cells and induced apoptosis in tumor cells [249,250]. However, these inorganic nanoparticles-based delivery systems for miRNAs reported some challenges, such as lower loading efficacy, lower endosomal escape, and lack of cargo protection. Thereafter, polymer-based, and lipid-based nanoparticles-based platforms were utilized for miRNA delivery. In a study, cationic short polyurethane and branched polyethylenimine (PU-PEI)-based nanospheres containing miR-145 demonstrated significant downregulation of tumor growth in lung adenocarcinoma cells by inhibiting epithelial-mesenchymal trans differentiation [122].

The combination of PU-PEI-miR-145, radiotherapy, and cisplatin reduced the growth of metastatic tumors, indicating its promising role in miRNA-based cancer nanotherapeutics. Later, it was reported that the high molecular weight polyethylenimine (PEI), a high degree of branching, led to non-specific toxicity. Thereafter, researchers utilized low molecular weight PEI with a smaller degree of branching for miRNA delivery and demonstrated its efficient function. In an in vivo study, miR-145 and miR-33a mimics elevated programmed cell death and reduced tumor growth in colon cancer using low molecular weight polyethylenimine and suppressed the cancer cells proliferation [251]. The smaller degree of branching in low molecular weight polyethylenimine demonstrated reduced toxicities-associated issues which were otherwise observed with high molecular weight polyethylenimine.

The first miRNA-based cancer nanotherapeutics that entered clinical trials—Mirna Therapeutics—involve liposomes’ modified tumor suppressor miRNA (miR-34), termed as MRX34. MRX34 demonstrated promising results in phase 1 and phase 2 clinical trials in patients with hepatocellular carcinoma, renal cell carcinoma (RCC), and acral melanoma. Currently, five more miRNA-based cancer nanotherapeutics are currently in clinical trials either in phase 1 or phase II stage [244]. In the past few years, a combination approach employing the codelivery of miRNA, along with small molecule anticancer drugs, have indicated a superior therapeutic benefit in cancer nanotherapeutics. This combination approach provided several advantages over conventional chemotherapeutics in inhibiting drug resistance, reversing epithelial to mesenchymal transition (EMT), inducing apoptosis and autophagy, suppressing tumor angiogenesis, and inhibiting overexpression of efflux transporters (P-glycoprotein) [252]. The targeted delivery of miRNAs combined with chemotherapeutic drugs sensitizes the cancer cells to chemotherapeutic drugs using an anti-miR system-based replacement or restoration of tumor genes [253]. Thus, the synergistic effect of the combinational therapy helps us to overcome drug resistance by directly targeting antiapoptotic signaling pathways and overexpressed efflux transporters. Shi et al. reported enhanced anticancer effects using lipid nanoparticles’ loaded miR-34 and paclitaxel drug compared to miRNA or paclitaxel alone [254]. Another study used polymer micelles coupled with miR-205 and gemcitabine to target markers such as OCT3/4, CD44, and Tubulin 3, showing a substantial reduction in tumor volume, implying that pancreatic cancer cells’ sensitivity to gemcitabine was restored [255]. Targeted co-delivery of miR-34a with anticancer drug in breast cancer displayed inhibition in chemoresistance, cell proliferation, and tumor invasion by modulating Notch-1 signaling pathway [256]. In a recent research work, the transfection of miR-126 mimic demonstrated an enhanced sensitivity of fourteen chemotherapy drugs (for example, trimetinib and alpelisib) through the inhibition of CDK4/6 and PIK3CA, which arrests cell cycle progression [257]. In another study, miR-1291 delivery along with gemcitabine and nab-paclitaxel to pancreatic cancer reported induced DNA damage, mitotic block, induced apoptosis, and significant inhibition of tumor cells growth by upregulating the AT-rich interactive domain-containing protein 3B (ARID3B) gene [229]. In another study, poly lactic acid and poly dimethylaminoethyl methacrylate conjugated with miR-21 inhibitor and doxorubicin (Dox) exhibited excellent anticancer efficacy in glioblastoma cancer cells [258]. Furthermore, research studies utilized the codelivery of miR-149 and miR-137 along with Dox to target neuroblastoma and pancreatic cancer cells and indicated restrained cell proliferation, promotion of apoptosis and sensitivity towards anticancer drug [259,260]. In a recent study, the injection of lipid nanoparticles conjugated with miR-634 and drug displayed induced apoptosis and reduced tumor growth in pancreatic cancer cells [261]. Although nanoparticles-mediated miRNA delivery has shown immense potential in the past few years, still, specific uptakes by cancer cells remain challenging due to the broad specificity of miRNAs. To overcome this challenge, nanoparticles are coated with either specific antibodies or ligands which are specifically expressed in cancer cells for targeted delivery. In a research study, polymeric micelles were conjugated with I-131-labeled prostate-specific membrane antigen (PSMA) antibody and demonstrated the co-delivery of miRNA and chemotherapeutic drugs to prostate cancer cells without any adverse effects [262]. Furthermore, nanoparticles conjugated with aptamers also showed promising results in the co-delivery of miRNA and drugs with enhanced cytotoxic activity against cancer cells [263,264]. Taken together, the combinational strategy by co-delivering anti-tumor miRNAs with chemo drugs synergistically enhanced the therapeutic efficacy with the reduction of cancer drug resistance. These studies signify that this approach would provide a research direction and various hopeful avenues for cancer therapies.

### 4.5. Self-Assembly Prodrug (SAP)-Based Nanotherapeutic Strategies

Conventional chemotherapy using anticancer medicines has several limitations, including low solubility, bioavailability, and, most crucially, MDR. To address the limits of free pharmaceuticals, a strong and effective nanotherapeutic technique, the self-assembling prodrugs-based approach, has emerged as a promising treatment option for cancer. This approach offers a strong and successful nanotherapeutic technique that received much attention in the past few years for the targeted delivery of poorly soluble anti-cancer medicines. SAP nanotherapeutics (SAPNS) are a very well-designed method, with various inherent benefits over free drugs that were previously clinically unmet by traditional approaches. SAPNs have better physicochemical qualities in terms of solubility, drug loading, chemical stability, and blood circulation. Second, they have better pharmacodynamic characteristics that favorably alter PK, drug release, and tumor uptake, while minimizing adverse effects. Third, this approach reduces systemic non-specific toxicities and serves as an effective carrier for the targeted administration of poorly soluble drugs in vivo. Fourth, the greater medication accumulation at the tumor site of the targeted delivery based on the enhanced permeability and retention (EPR) effect is a factor to consider [265,266]. Additionally, this SAPNs-based strategy utilizes a nanoparticle-mediated endocytosis cellular absorption mechanism, which aids in bypassing MDR-related issues. This endocytosis-mediated cellular absorption process circumvents drug efflux transporters, which are known to pump out free drugs.

After the last two decades, the self-assembling prodrugs (SAP) method has attracted considerable attention as a strong therapeutic platform for the enhancement of targeted tumor treatment [266,267,268,269]. SAPNs are classified into three types: lipid-drugs, polymer-drugs, and drug-drug conjugates [270]. Earlier research largely used hydrophilic polyethylene glycol (PEG) for combination with lipophilic medicines due to its ease of formulation, high hydrophilicity, and biocompatibility, which allowed for the avoidance of solubility and bioavailability difficulties associated with free drugs [271,272]. PEG-based prodrugs do not only self-assemble to different nanoformulations, such as polymeric micelles, but also provide synergistic anti-cancer activity by co-delivering the water-insoluble chemotherapeutics incorporated in their hydrophobic core [273,274]. Thereafter, another robust strategy using lipid-based modification emerged for the formulation of hard-to-formulate drugs by facilitating their self-assembly into nanoparticles of different shapes [275,276]. In a study, doxorubicin (DOX)-derivatized α-d-tocopherol succinate prodrug (N-DOX-TOS) and were able to form nano-assembly in aqueous solution after stabilization with TOS and demonstrated improved anticancer efficacy compared to unmodified DOX [277]. In another study, self-assembling doxorubicin prodrug PEG_2K_-DOX demonstrated their effective reversal of doxorubicin related drug resistance with enhanced plasma pharmacokinetics and in vivo therapeutic efficiency against MDR xenograft tumors when compared to doxorubicin alone [278]. Yang et al. reported an improvement in the sensitivity of cisplatin to triple-negative breast cancer using platinum Pt (IV) prodrugs based on cisplatin and chemosensitizer adjudin (ADD), which havw ability to self-assemble into nanosheets. This Pt (IV)-ADD-based self-assembled prodrug nanotherapeutics indicated an improved in vivo tumor growth inhibition with 266-fold lower IC_50_ value [279]. In a recent study, a synergistic Pt (IV) prodrug, Npx-pp-Pt (IV) demonstrated dual responsive behaviors for deactivating the dual drug resistance-related pathways to reverse cisplatin resistance. Herein, the in situ supramolecular self-assembly of prodrug into nanofiber structure revealed the enhanced cellular uptake of cisplatin and significant damage of the cisplatin-resistance cancer cells through cyclooxygenase-2 and nuclear factor kappa B-mediated apoptosis pathways, with a 80% tumor inhibition rate [269]. Furthermore, by exploiting the unique physicochemical properties of different drugs, amphiphilic drugs (hydrophilic drug conjugated with hydrophobic drug) can self-assemble into various nanoparticles shapes with improved pharmacokinetics, bioavailability, and antitumor efficacy [280]. In the co-delivery-based combination cancer therapy, different drugs are physically loaded in different nanocarriers. However, no physical drug loading is required with the drug–drug conjugate approach, as it already contains two distinctly pharmaceutically active agents [265,281]. Moreover, the self-assembled prodrug nanotherapeutics approach utilizes a drugs cocktail that alleviates the nonuniform biodistribution of anticancer agents and also ensures well-controlled targeted dual-drug delivery to reverse multi-drug resistance in cancer therapeutics.

### 4.6. Exosomes-Based Nanotherapeutics Strategies

Exosomes represent a subclass of heterogeneous extracellular vesicles (EVs) of endosomal origin with a diameter of 40–150 nm, which are secreted from a variety of cells present in tumor microenvironment such as cancer cells, tumor associated fibroblasts, CSCs, and tumor associated immune cells [282]. In the tumor microenvironment, exosomes-mediated constant crosstalk between tumor cells and stromal forms a large part of the communication. Exosomes are involved in various cellular and pathological conditions and, through intercellular communication, deliver their cargo to the immediate surroundings, as well as at distant organs. The cargo of exosomes comprises proteins, lipids, nucleic acids, and metabolites that modulate stromal reactions, regulates immune response, promotes angiogenesis, and modify signaling pathways related to cancer in tumor microenvironment. Numerous in vitro and preclinical in vivo studies demonstrate that exosomes play a critical role in conferring drug resistance on cancer cells via intercellular interactions in a variety of cancer types, including pancreatic cancer, breast cancer, lung cancer, prostate cancer, colorectal cancer, glioblastoma, kidney cancer, neuroblastoma, ovarian cancer, gastric cancer, melanoma, and osteosarcoma [283,284]. Exosomes’ cargo mediates chemoresistance through the regulation of drug efflux and metabolism, epithelial–mesenchymal transition, alteration of prosurvival signaling pathways, remodeling of tumor microenvironment, and increase concentration of plastic CSCs [285]. Along with their crucial involvement in establishing drug resistance in cancer, exosomes also transmit drug resistance phenotypes to other cancer cells and serve as biomarkers for monitoring drug resistance in cancer. Exosomes, by virtue of their function in chemoresistance, might also be used as a therapeutic target for overcoming drug resistance in cancer cells.

In order to enhance the effect of chemotherapy, exosome-mediated chemoresistance inhibition is prerequisite. In this context, two possible strategies are available that include exosome biogenesis and trafficking suppression, depletion of exosome uptake by cancer cells, modulation of harmful exosomal cargo, and inhibition of exosome dissemination, removal of exosomes. Exosomes depletion and removal may restore drug sensitivity to chemotherapy to some extent. However, limited knowledge regarding the specific ways how exosomes are internalized by cancer cells and deliver their cargo pave the way for alternative strategies to overcome drug resistance. Therefore, the application of exosomes as drug and gene delivery vehicles for targeted cancer nanotherapeutics is an appealing platform owing to its natural composition, low toxicity, and low immunogenicity. In cancer nanotherapeutics, different synthetic nanoparticles such as liposomes, self-assembling peptides and nanosponges were extensively utilized for targeted cancer therapy [286,287]. Nonetheless, various challenges such as different biological barriers due to the tumor heterogeneity still remain, with the exogenous nanomaterials being utilized for targeted drug delivery to cancer cells [288]. To overcome the limitations of synthetic nanoparticles, one emerging approach is to develop and utilize natural nanocarriers for targeted delivery. Several intrinsic features of exosomes, such as the ability to pass through lipid bilayer of cell membrane, high delivery efficiency, good stability in biological fluids, and high biocompatibility with low immunogenicity, support their potential as attractive nanocarriers for targeted drug or gene delivery [289,290]. Their specificity may further be improved upon by engineering exosomes with tumor-specific peptides, proteins, or antibodies for precise targeted drug delivery. The critical steps involved in utilizing exosomes as nanocarriers are the development of an efficient cargo loading method and choice of exosome-producing cells as these steps greatly impact the function, biodistribution, and immunogenicity of the exosomes. The exosomes-loading approaches include passive diffusion; electroporation, and loading the cargo to parental cells by incubation, overexpression, or transfection; and isolation of secreted exosomes through extrusion, freeze and thaw cycles, and sonication [291]. Regarding cell types, cells should be selected which are scalable and can produce large quantities of exosomes such as mesenchymal stem cells (MSCs) and bovine milk [292,293].

Exosomes loading with small molecule chemotherapeutic drugs attracted much attention in the past few decades. Researchers obtained paclitaxel-loaded exosomes from the centrifuged supernatant of chemo-resistant cells treated with paclitaxel. The supernatant contained drug loaded exosomes as the chemo-resistant cells natural tendency to flush out the drugs due to overexpression of drug efflux transporters [294]. Nevertheless, drug loading in exosomes demonstrated improved bioavailability, stability in biological fluids, and reduced off target effects. In this line, paclitaxel loaded exosomes increased the toxicity by 50-fold in drug-resistant cells by ensuring co-localization of exosomes carrier with cancer cells [295]. Despite encouraging results using exosomes as drug delivery vehicles, still a few challenges remain such as purification, large scale production, and efficient drug loading and storage. Exosomes subgroups’ heterogeneity further slowdown the quality control processes and translation into clinical settings [284]. Therefore, the development of artificial exosomes through te advancements in nanobiotechnology opens several avenues for advanced drug delivery.

The nano bioengineered artificial exosomes or exosomes mimics carrying anticancer drugs as drug delivery vehicles present the current pro-active approach in cancer nanotherapeutics. Jang et al. developed exosome mimics by mixing the doxorubicin drug with whole monocyte or macrophage cells followed by passage through filters of different pore sizes. The developed exosome mimics were compared with natural exosomes loaded with doxorubicin and indicated similar properties, but a 100-fold higher production yield [296]. Several preclinical studies utilized exosomes-based delivery approach for the targeted delivery of paclitaxel and doxorubicin to different cancer types, such as prostate, pancreatic, and lung cancer [293,297]. The results reported superior delivery of drugs through exosomes as compared to liposomes and free drugs. Kim et al. demonstrated exosomes-based successful delivery of paclitaxel to MDR cancer cells with overexpression of efflux transporters P-glycoprotein (P-gp). Paclitaxel loaded exosomes indicated the reversal of drug resistance by providing enhanced sensitivity towards MDR cancer cells by escaping P-gp-mediated drug efflux and inhibiting metastasis in a lung cancer xenograft model [295]. In a similar approach, gold nanoparticles’ conjugated doxorubicin was loaded into exosomes and displayed an improved antitumor effect against lung cancer cells [298]. Furthermore, exploration of exosomes content escalated a vital role in the reversal of chemoresistance as they have a direct role in the development of chemoresistance [299]. Wu et al. (2020) utilized exosomes derived from bone marrow mesenchymal stem cells loaded with miR-193a for targeting leucine rich repeat and revealed reduced cisplatin resistance in non-small cell lung cancer [300]. In another study, engineered exosomes were employed for co-delivery of miR-21 inhibitor 5-fluoro-2,4(1H,3H)-pyrimidinedione(5-FU) for the reversal of drug resistance in colon cancer via targeted chemotherapy [51]. Shtam et al. showed a reduced level of DNA damage-repair protein and induction of apoptosis levels using exosome loaded with RAD51 siRNA in fibrosarcoma and cervical adenocarcinoma cell lines [301]. In a similar approach, exosomes derived from fibroblasts loaded with kras-siRNA indicated superior delivery and blunted tumor growth in pancreatic cancer [302]. In another study, exosomes isolated from HEK-293 cells were transfected with HGF siRNA demonstrated reduced vascularization with reduction in levels of HGF and VEGF proteins in gastric cancer cells tumors compared to free siRNA [303]. Apart from siRNA, miRNA was also loaded within exomes for targeted delivery and inhibition of tumor growth. Several studies reported improved nanotherapeutics using exosomes-loaded miRNA delivery (miR-143, miR146b, and miR-122) to human osteosarcoma cells, glioma cells, and hepatocellular carcinoma (HCC) cells, respectively [290,304,305]. Adipose tissue-derived MSCs (AMSCs) released exosomes transfected with miR-122 induced sorafenib chemosensitivity when co-cultured with hepatocyte carcinoma cells [304]. In a similar approach, co-culture exosomes derived from AMSCs carrying miR-199a induced chemosensitivity towards doxorubicin by downregulating mammalian target of rapamycin (mTOR) pathway and [305]. Furthermore, Kim et al. reported inducted apoptosis and cisplatin chemosensitivity using exosomes loaded with CRISPR/Cas9 and si-c-Met in ovarian cancer cells and human gastric adenocarcinoma cells, respectively [306,307]. Oxiplatin-resistant cancer-resistant cells demonstrated chemosensitivity and decreased motility with normal intestinal FHC cell-derived exosomes loaded miR-128-3p [308]. In recent research reports, induced chemosensitivity towards trastuzumab and docetaxel were reported in breast cancer cells and tongue squamous cell carcinoma through exosomes-mediated delivery of miR-567 and miR-200c, respectively [309,310]. Moreover, exosomes-mediated targeted delivery holds promising strategy for reversing chemoresistance by delivering conventional drugs and various genetic materials. Overall, exosomes-based targeted delivery of drugs and genes are a new and emerging approach which holds much promise for drug resistance reversal. However, further exploration of the different sources of exosomes, side effects, and safety would be pertinent for cancer nanotherapeutics in order to attain higher delivery efficacy for anticancer molecules at lower doses without any side effects.

## 5. Clinical Trials and Update

Despite the potential and promising results indicated by nanoparticulate targeted systems in pre-clinical studies, only a few cancer nanotherapeutics-based strategies are translated to clinical settings. The challenges associated with targeted nanoplatform to reach clinical settings include several barriers such as efficacy, safety, scalability, regulatory issues, and lack of much resemblance of pre-clinical models with human tumors [115,311]. The prominent model nanosystems that either reached clinical trial stage or were approved by drug regulatory bodies include liposome, polymer nanoparticles, lipid-based nanoparticles conjugated with siRNA, miRNA, and the polymeric micellar nanoparticulate system [32,312,313]. Currently, nine nanoparticulate-based cancer therapies are approved by the Food and Drug Administration (FDA) for therapeutics and about 30+ more nanomedicine-based systems in clinical trials [314]. Some of the prominent nanotherapeutics which are in various developmental stages of clinical trials or were approved by FDA are listed in Table 6.

Currently, liposomal formulations are dominant under clinical evaluation among the nanoparticulate targeted systems currently under preclinical development or in clinical trials. The first FDA-approved nanoparticulate nanomedicine doxil^R^/Caelyx^R^ (doxorubicin) in 1995 was liposomal formulations [37,315,316]. The clinical benefits so far observed with liposomes-based doxil^®^ include reduced toxicity, rather than improved efficacy [315,317]. Afterwards, other approved liposomal formulation Vyxeos^R^ (daunorubicin/cytarabine) reported improved response rate and survival rate in phase III clinical trials of therapy-related acute myeloid leukemia (t-AML) patients [318]. Furthermore, albumin nanoparticles-bound paclitaxel, Abraxane^®^, is now approved in more than 40 countries for the treatment of metastatic cancers (breast cancer, non-small cell lung cancer, and pancreatic cancer) [319]. Both doxil^R^ and Abraxane^®^ were commercially successful, with a huge market over the past few years. The global market for liposomal doxorubicin was reported to be more than USD 1100 million in 2021, and is expected to grow further by the rate to reach over USD 1569 million by 2027 (https://www.expertmarketresearch.com/reports/liposomal-doxorubicin-market) (accessed on 15 November 2021). Likewise, the Abraxane^®^ market size is currently around USD 254 million in 2022 and is reported to increase with a growth rate of 2% each year for pancreatic cancer and lung cancer worldwide [320]. Apart from these abovementioned FDA approved products, some other approved products include Ontak^®^ (denileukin diftitox for cutaneous T-cell lymphoma treatment), Onivyde^®^ (liposomal irinotecan for pancreatic cancer treatment), Nanoxel M (paclitaxel for ovarian and pancreatic cancer treatment), Genexol PM (paclitaxel for metastatic breast cancer and lung cancer treatment), Myocet^®^ (doxorubicin for metastatic breast cancer treatment), and DepoCyt^®^ (liposomal cytarabine for enhanced tumor targeting) represent some of the products which are FDA-approved and commercially available for clinical use in cancer patients [36,314].

Currently, the majority of clinically approved nanotherapeutics-based strategies utilize a passive targeting approach to deliver drugs or therapeutic agents, for example, doxil^®^ and DaunoXome^®^ employ non-targeted liposomes as their carrier [321]. However, there are limited clinical trials ongoing with the targeted delivery strategy over the last 40 years. To our knowledge, only two active nanoparticle targeted products (albumin nanosphere and liposomes-based product) are approved by the FDA and commercially available in market. One of the factors contributing to the limited success of the targeted cancer nanotherapeutics clinical settings include lack of knowledge about tumor heterogeneity, which ultimately affects the intratumoral nanoparticle penetration. Other factors include safety, efficacy, and scalability of nanoparticulate-based systems along with regulatory issues. These limitations provide better opportunities to nanotechnologists and materials scientists to further investigate the correlation between physicochemical properties of nanoparticles and their integration with tumor microenvironment components. These nanoparticles and tumor microenvironment interactions should be conducted in suitable murine or human models. In order to accelerate the clinical translation systematic investigation results should be utilized for the development of mathematical models for accurate predictions. Nevertheless, nanoparticles alone or in combination with different nucleic acids present a ray of hope for improved cancer therapy with higher survival in clinics, with a better understanding of tumor microenvironment (both cellular and non-cellular components) and tumor heterogeneity in future.

**Table 6:** An update of nanoparticulate nanomedicine-based anticancer therapeutics clinical trials studies.

Nanoparticulate System	Drug/Therapeutic Agent	Type of Cancer	Findings	Clinical Trials Status	Reference
Liposomes	Doxorubicin	Primary and metastatic liver cancer	Well tolerated by patients (*n* = 18) with 33% response rate	FDA-approved	[322]
Albumin nanoparticles	Paclitaxel	Squamous cell carcinoma	Well tolerated by patients (*n* = 42) with 81% response rate	FDA-approved	[323]
Liposomes	Cisplatin	Advanced malignant tumors	51% clinical benefit with 11.1% partial response in patients (*n* = 12)	Active, phase II clinical trials	[324]
PEG and polyaspartate polymeric nanoparticles	Paclitaxel	Bile duct, pancreatic, gastric and colon cancer	30% stable disease and 5% responded well (*n* = 19)	Active, phase III clinical trials	[325]
Liposomes	Vincristine sulphate	Acute lymphoblastic lymphoma	22% complete and partial response (*n* = 36)	FDA-approved	[326]
Albumin nanoparticles (ABl-007)	Nanoparticle bound paclitaxel and free gemcitabine	Metastatic breast cancer	Well-tolerated and 81% response rate (*n* = 42), 8% complete response, and 42% complete response (*n* = 50)	FDA-approved	[327]
NK012 polymeric nanoparticles	SN-38 (Camptothecin analogue)	Solid tumors	9% partial response (*n* = 11)	Active, phase II clinical trials	[328]
Immunoliposomes	Doxorubicin and anti-EGFR	Advanced solid tumors	38% stable disease, 8% complete and partial response (*n* = 26)	Active, phase II clinical trials	[329]
Liposomes	Annamycin	Acute lymphoblastic leukemia	16% partial response (*n* = 31)	Active, phase II clinical trials	[330]
Liposomes	Vincristine sulphate	Acute lymphoblastic lymphoma	41% complete and partial response (*n* = 56)	FDA-approved	[331]
PEP02 liposomes	Irinotecan and Docetaxel	Gastro-esophageal adenocarcinoma and metastatic gastric	14% complete and partial response (*n* = 44)	FDA-approved	[332]
Polymeric CRLX101 nanoparticles	Camptothecin	Advanced solid tumors	64% stable disease (*n* = 44)	Active, phase II clinical trials	[333]
Lipid nanoparticles	VEGF and KSP siRNAs	Advanced solid tumors	42% stable disease (*n* = 24)	Limited progression of siRNAs into phase II	[240]
Cationic liposomes	wt human p53 plasmid	Advanced solid tumors	64% stable disease (*n* = 11)	Active, phase II clinical trials	[334]
Bind-014 coated nanoparticles	Docetaxel	Advanced solid tumors	12% complete and partial disease response (*n* = 52)	Active, phase I clinical trials	[335]
Lipid core nanoparticles	Paclitaxel	Epithelial ovarian sarcoma	43% progression free survival (*n* = 14)	Active, phase II clinical trials	[336]
NC-6004 micellar nanoparticles	Cisplatin	Advanced solid tumors	70% stable disease and 15% partial response (*n* = 22)	Active, phase III clinical trials	[337]
PEG protein conjugate	L-asparaginase	Lymphoblastic leukemia	77.8 complete response and 3.7% partial response, Overall survival of 50% or better (*n* = 162)	FDA-approved	[338]
PEG polymer micelles	Epirubicin	Advanced and recurrent solid tumors	53% stable disease and 5% partial response (*n* = 47)	Terminated (did not cross after phase I trials)	[339]
Liposomes	MRX34 (miR-34a)	Advanced solid tumors	13% stable disease and 68% partial response (*n* = 47)	Terminated	[340]
Activated carbon nanoparticles	Epirubicin	Breast cancer	No response	Terminated	[341]
DOTAP-cholesterol nanoparticles	TUSC2 plasmid	Lung cancer	23% partial response and stable disease (*n* = 31)	Terminated (did not cross after phase I trials)	[342]
CYT-6091 colloid PEGylated nanoparticles	Recombinant human TNF-α	Solid organ cancer	1% complete and partial response (*n* = 156)	Terminated (did not cross after phase I trials)	[324]
Rexin-G nanoparticles	Cytocidal cyclin G1 construct	Sarcoma and osteosarcoma	88% stable disease or partial response (*n* = 17)	Terminated (did not cross after phase II trials)	[343]

## 6. Challenges and Future Prospective

In the past few years, cancer nanotherapeutics present an emergence and proved to be an effective strategy for the enhancement of anticancer chemotherapeutics and minimizing toxicity for overcoming cancer drug resistance and recurrence. Nanotherapeutics-based approaches using different types of nanocarriers show great potential in the development of various targeted therapies for treating wider range of human cancers. The nanocarriers utilized in cancer nanotherapeutics include liposomal nanoparticles, metal nanoparticles, polymeric nanoparticles, polymeric micelles, dendrimers, exosomes, nanogel and others to targeted the delivery of drugs and genes. At the preclinical and clinical stages, both new drugs and newer nanoparticulate systems-based strategies are developed for targeted delivery to tumor site. However, the cancer nanotherapeutics field is relatively new and ever-evolving wherein the exploration of different types of nanocarriers/nanoparticulate systems and their efficacy evaluation are still needed. Despite tremendous developments in cancer nanotherapeutics, there are still a number of difficulties that must be overcome before it may be used in clinical settings [17,34]. Currently, only nine nanoparticles-based products are in market and 30 plus in clinical trials for cancer therapy despite numerous pre-clinical trials performed or on going. The main challenges responsible for the poor clinical translation using nanomaterials-based strategies include characterization and reproducibility problems, limited bioavailability, retention in macrophages of reticuloendothelial system, toxicity/safety issues, less knowledge about tumor heterogeneity, efficacy changes from animal to human models, scale-up challenges, economic challenges, and regulatory consideration related to approval [37,39,311,344]. Therefore, in order to make this more efficient, cost-effective, and patient compliant, nanocarriers-mediated targeted strategies, various physiological, technological, and regulatory barriers need proactive consideration. The technical challenges include the formulation and characterization feasibilities of the nanoparticulate system, while the physiological challenges include consideration related to correlations between in vitro and in vivo findings. Considerations for the approval of nanomedicine-based tailored techniques for clinical use and other regulatory difficulties are among the hurdles. In the past few decades, both active and passive targeting methods were used for cancer therapy in order to enhance the quality of life and overall survival of patients, and some of these tactics were also adapted to clinics. Among all the FDA-approved nanoparticulate products, only two products are based on active targeting and most of them belong to the passive targeting approach. In the passive targeting strategy, EPR effect plays a vital role in targeting specific tumor site. However, there is a great variability of the EPR effect, not only with the tumor vasculature, but also with the tumor types and tumor models [345]. Thus, the prediction of clinical efficacy based on pre-clinical studies data seems tricky, as only 8% of animal studies translate into clinical trials [346]. As human physiology and biochemistry are drastically different from other small animals in various ways, the lack of efficacy poses most common challenge in translating to human system despite its promising results in different animal models viz. the patient-derived tumor explant model or genetically engineered mouse models. In order to alleviate this challenge, an evaluation of EPR activity in each specific patient using nanoparticles-based diagnostics is necessary in clinical practice. Furthermore, in order to enhance the EPR effect co-administration of agents to augment tumor vasculature or active tumor targeting may prove potential approach to overcome those shortcomings [345,347]. Advanced in vitro tumor models such as genetically engineered, orthotopic, and metastatic tumor models with through characterization should provide more accurate indications. These in vitro tumor models would save the time and unnecessary animals usage. These models would screen different therapeutic drugs/agents for their effectiveness determination and ultimately are verified in large animal models in a hierarchal approach that allows us to provide more authentic data for further clinical trials.

Currently, novel methodologies are being designed for drug/gene-based targeting of CSCs and specific tumor microenvironment components responsible for the tumor survival and intrinsic drug resistance, representing major directions in drug-resistant therapeutics [12]. In this approach, better tactics for modulating the tumor microenvironment cellular and non-cellular components, the pro-survival signaling pathways and expression of drug efflux transporters employing various nanocarriers and polymer-drug conjugates are practiced with enhanced efficiency. The rational design of novel effective and safer nanomaterials will necessitate a thorough knowledge of nano-biointeraction (interactions between nanoparticles and the tumor microenvironment). In the current state of cancer treatment, a variety of new and innovative strategies, such as nanotherapeutics that target cancer stem cells, siRNA-based nanotherapeutics, miRNA-based nanotherapeutics, self-assembling prodrugs, and exosomes-mediated nanotherapeutics, are being used to actively target the tumor microenvironment [17,201,269,307]. These nanomaterials interact with intracellular structures, biological barriers, blood components, and the cell membrane, which is why nanotechnologists and bioinformaticians are developing predictive models in silico to better understand these interactions [348]. Computational toxicology models, macromolecular pharmacokinetic model simulations, and other simulation approaches such as imaging procedures help us attain a better knowledge of nanobiointeractions. Researchers might use this knowledge to create more targeted nanoparticle-mediated drug delivery systems and make a more informed decision [349,350].

The instability of nanocarriers, their dysregulated accumulation within cancer cells, and the development of multi-drug resistance make it difficult to precisely target cancer cells using nanomaterials. Drug resistance in cancer treatment may be addressed by using nanomaterial-based drug carriers adorned with combinations of drugs/therapeutic agents for tumor site-specific chemotherapy and other combinations/synergistic treatments [351]. Nanoparticle drug carriers-based combination therapy needs further studies at the preclinical and clinical levels. Furthermore, multifunctional nanoparticles also offer alternative option to improve biodistribution, localization, and efficacy of drugs in order to meet precision cancer diagnosis and therapy. In the past few years, the sophisticated design of nanoparticulate system encompassing the ability to deliver multimodal therapeutic agents for providing synergistic cancer therapies with reduced dosage requirements and toxicities have escalated much attention. Improved nanomaterials design provides a multifunctionality feature for therapeutic encapsulation and specific tumor targeting. Moreover, multifunctional nanomedicines serve as therapeutic cargos for multi-drug combination therapy with the ability to co-deliver or multimodal combination therapy to surmount MDR. Advanced nanotherapies are targeting the drug-resistant CSCs using sensing therapeutic drugs capable of modulating signaling pathways, proapoptotic proteins or P–gp inhibitors which promote the mitotic-inactivation of CSCs [43].

The foremost challenge and limiting factor currently of paramount concern in cancer nanotherapeutics is the safety profile of nanoparticles for clinical translation. Metal nanoparticles were extensively utilized in cancer therapy; however, toxicity issues hinder their progress towards clinics [37]. Thus, there is an urgent need to overcome the toxicity-related effects associated with nanomaterials. To attempt this, alteration of nanoparticles physiochemical properties through polymer modifications and increased usage of ligand attachment at the pre-clinical stage of development is being carried out. Thereafter, the toxicity assessment of nanoparticles must be performed at the clinical stages (phase I, phase II, phase III, etc.) to use nanoparticles as an excipient for human use [314]. In cancer nanotherapeutics, lipid and lipid-based nanoparticles are commonly utilized for drug/gene delivery due to their non-toxicity. In the past few years, naturally derived exosomes or endogenously biocompatible lipids, such as LDL and HDL (low and high-density lipoprotein, respectively) are utilized to ensure safety and deliver the therapeutic agents to cancer cells [352,353]. Some of the LDL and HDL nanoparticles-based systems are already in phase I and II clinical trials without causing any toxicity issues at any dosage levels [352]. However, naturally occurring nanocarriers face scalability issues for clinical trials and require alternative strategies. In this context, naturally, biomaterials are modified for their enhancement of the stability, pharmacodynamic, and pharmacokinetic properties, but require thorough investigation. To become a good excipient for clinical trials, nanoparticles must be quickly eliminated from the system and, in the event of gene delivery with poor transfection, must be easily cleared from the system. The clearance of nanomaterials, either via the liver into the bile duct or through the kidney into the urine, should be the primary criterion before clinical translation, since their accumulation may create further problems [354]. This is further complicated by the fact that nanoparticles which promote higher cellular uptake are at greater risk of producing cytotoxicity or incomplete clearance in vivo. Therefore, synergy analysis is required in the case of combination therapies to ensure low loading requirement by the nanocarriers through optimization of therapeutics.

Rosenblum et al. discussed several other limitations related to targeted delivery of cancer nanotherapeutics which limit its clinical success translation. The limitations include tumor heterogeneity with different morphological and phenotypical tumor profiles, tumor penetrability of nanoparticles, relatively hypoxic microenvironment, and endosomal escape [311]. To overcome the endosomal escape issue, naturally derived exosomes derived from milk or stem cells or bioengineered exosomal mimics with ability to escape endosomal pathway are being employed for targeted delivery. In a recent report, transport across tumor endothelium poses a major challenge in cancer nanotherapeutics in terms of nanoparticles penetration and diffusion and further accumulation inside tumors [344]. Viscous tumor interstitium, along with permeability, nonlinear diffusion rates, tumor flux, marginal efficiency of blood vessels and unpredictable blood flow rates inside the tumor, niche put forward additional challenges [37]. Another challenge associated with cancer nanotherapeutics include economic risk as the preclinical and clinical trials using nanomaterials are expensive and time consuming. To overcome these shortcomings and uplift the country’s economy, efforts are being taken to develop an effective collaboration between different research laboratories, clinicians, public initiatives, and investors to apply risk mitigation schemes for reduction of overall coast of the projects. Furthermore, additional regulatory difficulties such as product registration and regulatory norms necessary for FDA/other-approved administering bodies’ basic criteria for preclinical laboratory evaluations must be considered.

## 7. Conclusions

To overcome or reverse drug resistance, cancer nanotherapeutics have shown a promising therapeutic alternative to existing cancer therapies. However, these agents require more characterization and optimization before they can be used in clinical trials. However, with the rapid development of nanotechnology and materials research, there are toxicity and efficacy issues that suggest better understanding of the tumor microenvironment, development of newer strategies (CSCs targeting, nucleic acids delivery, self-assembly prodrugs, exosomal delivery), and clinical trials using nanoparticulate-based systems. In addition, nanomedicine formulations with increased efficacy and lower toxicity should receive adequate attention in large-scale commercial batches that are reproducible. Taken as a whole, the development of nanoparticulate systems should be focused on their capacity to reach the clinical setting, as well as commercialization.

## Figures and Tables

**Table 1:** A representative list showing different mechanisms along with drugs, molecular targets, and cancer type associated with cancer drug resistance.

Resistance Mechanism	Cytotoxic Drugs	Type of Cancer	Target	Reference
miR-27 involved resistance	Platinum drugs, Doxorubicin	Esophageal cancer	Micro-RNA 27a/b (miR-27a/b)	[44]
Microseminoprotein, prostate-associated (MSMP) gene upregulation	Vascular endothelial growth factor receptor 1/2/3 (VEGFR1/2/3) inhibitors	Ovarian cancer	Hypoxia, triggering Mitogen-activated protein kinases (MAPK) signaling	[45]
Activated PDGFR	Histone deacetylase inhibitors, phosphatidylinositol 3-kinase, anti-VEGF drugs	Prostate cancer	platelet-derived growth factor receptor (PDGFR)	[46]
Tumor heterogeneity	Tyrosine kinase inhibitors	Lung cancer	epidermal growth factor receptor (EGFR) T790M mutation	[47]
Tumor heterogeneity	Vemurafenib	Melanoma	Mutation in MAP kinase 1 (MEK1)	[48]
Drug inactivation	Platinum drug	Lung cancer	Thiol glutathione	[49]
Reduced drug uptake	Anthracyclines, axanes, oxazaphosphorines and platinum-based drugs	Breast cancer	Endocytic-mediated pathways	[50]
Reduced drug uptake	5-Fluorouracil (5-FU) and miR-21 inhibitor oligonucleotide (miR-21i)	Colon cancer	Micro-RNA-21 (miR-21)	[51]
DNA repair alternation	Olaparib	Prostate cancer	Poly (adenosine diphosphate [ADP]-ribose) polymerase (PARP)	[52]
DNA repair alternation	Platinum (carboplatin or cisplatin) and taxol (paclitaxel)	Ovarian cancer	DNA repair pathways	[53]
Inhibition in apoptotic pathways and autophagy	Epirubicin, tamoxifen, herceptin, and vinorelbine	Breast cancer	Autophagy	[54]
Epithelial to mesenchymal transition (EMT)	Wingless and Int-1 (Wnt) Signaling inhibitors	Ovarian cancers	Wnt/β-catenin signaling pathway	[55]
Epithelial to mesenchymal transition (EMT)	Nivolumab	Urothelial cancer	EMT/stroma-related gene expression	[56]

**Table 2:** Nanotherapeutic approaches to target cellular components of tumor microenvironment for overcoming cancer drug resistance.

Nanoparticles Platform	Targeted Component of TME	Drug/Therapeutic Agent/Surface Functionalization	Outcomes	Reference
Lipid-nanoparticle composite	Tumor-associated fibroblasts (TAFs)	Single chain tumor necrosis factor (TNF)	Enhancement of specific uptake and activity of TNF nanocytes	[120]
PEGylated carboxymethylcellulose nanocomposite	Tumor-associated fibroblasts (TAFs)	Docetaxel	Several fold increase in circulation time, and tumor perfusion, reduction in metastasis	[121]
Polyethyleneimine-β-cyclodextrin (PEI-β-CD) complex	Tumor-associated fibroblasts (TAFs)	CY11 peptide	Two-fold higher gene delivery efficiency	[122]
Gold nanoparticles	Tumor-associated fibroblasts (TAFs)	Fibroblast growth factor 1 (FGF1)	40% reduction in cell viability	[123]
Cleavable amphiphilic peptide (CAP) nanoparticles	Tumor-associated fibroblasts (TAFs)	Fibroblast activation protein-α (FAP-α)	Disorganization of the stromal barrier, enhancement of local drug accumulation	[124]
Nanoparticle-based photoimmunotherapy (nano-PIT)	Tumor-associated fibroblasts (TAFs)	Fibroblast-activation protein (FAP)	Significantly enhanced T cell infiltration, and efficient tumor suppression.	[125]
Antibody-drug conjugate (ADC)	Tumor-associated fibroblasts (TAFs)	Tumor endothelial marker 8	Blocked metastatic growth, and prolonged overall survival.	[126]
Conjugated nanoparticulate system	Tumor-associated fibroblasts (TAFs)	Cisplatin, siWnt16	Knockdown of Wnt16	[127]
Poly (lactic-co-glycolic acid) (PLGA)	Tumor-associated fibroblasts (TAFs)	Rapamycin	Modulation of tumor vasculature	[128]
Nanohydrogel particles and lipoplexes	Tumor-associated fibroblasts (TAFs)	Cyclic peptide and siRNA	Enhanced in vivo uptake, functional siRNA delivery	[129]
PLGA nanoparticles conjugated with Arginine-glycine-aspartic acid (RGD)	Tumor-associated vascular endothelial cells	Paclitaxel (PTX) and combretastatin A4 (CA4)	Tumor vasculature disorganization, inhibition of cell proliferation, significantly enhanced apoptosis	[130]
PEG-PLA nanoparticles	Tumor-associated vascular endothelial cells	F3 peptide	Deep penetration at the tumor side, Enhanced accumulation with longest survival	[131]
Nanographene oxide nanocomposite	Tumor-associated vascular endothelial cells	TRC105, a monoclonal antibody that binds to CD105	Improved uptake at tumor site	[132]
Polyacrylic acid (PAA)-coated superparamagnetic iron oxide	Tumor-associated vascular endothelial cells	RGD	Tumor targeting and antiangiogenic response	[133]
Cholesterol-based nanoparticles	Tumor-associated vascular endothelial cells	Doxorubicin (Dox) and RGD	15-fold increase in antimetastatic activity	[134]
Gold nanorods	Tumor-associated vascular endothelial cells	RGD	Downregulation of integrin α(v)β₃ expression	[135]
PEG nanoparticles	Tumor-associated macrophages (TAMs)	Mannose	Efficient targeting of TAMs	[136]
Polymer nanoparticles	Tumor-associated macrophages (TAMs)	Mannose and siRNA	Enhanced uptake and efficient delivery of siRNA	[137]
PLGA nanoparticles	Tumor-associated macrophages (TAMs)	Antigenic peptides, hgp100 (25–33) and TRP2 (180–188)	Significantly delayed growth of melanoma	[138]
PLGA-based nanoparticles	Tumor-associated T cells	Inhibitor of transforming growth factor beta receptor 1 (TGFβR1)-R848	Promotes infiltration of T cells, improved efficacy for delivery	[139]
PLGA-based nanoparticles	Tumor-associated antigen presenting cells	anti–PD-1 monoclonal antibodies	Increase in expression of adhesion molecules, enhance antitumor immunity	[140]
Lipid-coated calcium phosphate nanoparticles	Tumor-infiltrating T-lymphocytes	siRNAs against PD-1 and PD-L1	Effective delivery of siRNAs, silencing of PD-1 and PD-L1 expression, improved cytotoxicity	[141]
Poly(lactic-co-glycolic) acid (PLGA) nanoparticles	Tumor-infiltrating T-lymphocytes	Indocyanine green (ICG), imiquimod (R837)	Checkpoint-blockade, effective immunotherapy	[142]
Polymer nanoparticles	Tumor-associated leukemia-specific T cells	DNA	Effective targeting of chimeric antigen receptors (CARs), long-term disease remission	[143]
Liposome nanoparticles	Tumor-infiltrating lymphocytes (TIL)	Antagonist for the adenosine receptor A2A (SCH-58261)	Controlled drug effects on cells, enhanced active targeting	[144]
TH10 peptide nanoparticles	Tumor-associated pericytes	Docetaxel	Pronounceable pericyte apoptosis induction	[145]
Liposome nanoparticles	Tumor-associated lymphatic vessels	Doxorubicin, cyclic peptide (LyP-1)	Increased liposome uptake, reduction in metastasis	[146]

**Table 3:** Nanotherapeutic approaches to target non-cellular components of tumor microenvironment for overcoming cancer drug resistance.

Nanoparticles Platform	Targeted Component of TME	Drug/Therapeutic Agent/Surface Functionalization	Outcomes	Reference
Sorafenib (Sor) nanoparticles	Tumor hypoxia	Apoptosis inducer (CA IX-C4.16)	Synergistic therapeutic efficiency of CA IX-C4.16 and Sor combination	[147]
Terpolymer-Protein or protein-lipid nanoparticles	Tumor hypoxia	Manganese dioxide (MnO2)	Generation and delivery of different oxygen rates, 40% reduction in tumor growth in combination with radiotherapy	[148]
Carboxymethyl dextran nanoparticles	Tumor hypoxia	Doxorubicin and 2-nitroimidazole derivative	Selective accumulation of nanoparticles at hypoxic tumor tissues, high antitumor activity	[149]
Oxygen self-sufficient amphiphile (F-IR780-PEG) nanoparticles	Tumor hypoxia	Doxorubicin	Downregulation of P-glycoprotein expression, synergistic treatment by combination of chemotherapy and photodynamic therapy	[150]
CdTe quantum dots (QDs) conjugated with 2-deoxyglucose (DG)-polyethylene glycol (PEG), Lipoic acid, lysine, 9-poly-d-arginine	Tumor hypoxia	HIF-1α siRNA	Enhanced hypoxic tumor targeting, Excellent biocompatibility, perfect siRNA binding capability	[151]
Polyethylene glycol (PEG)-poly L-lysine (PLL)-poly lactic-co-glycolic acid (PLGA)-based nanoparticles	Tumor hypoxia	Transferrin (Tf) and daunorubicin (DNR)	Downregulation of HIF-1α expression, and induced apoptosis	[152]
Manganese ferrite nanoparticles	Tumor hypoxia	Mesoporous silica nanoparticles	Reduction in hypoxic environment with continuous O_2_-evolving property	[153]
Carboxymethyl dextran (CMD) and black hole quencher 3 (BHQ3) nanoparticles	Tumor hypoxia	Doxorubicin	Improved drug biodistribution, Enhanced toxicity under hypoxic conditions compared to normoxic conditions	[154]
Haemoglobin-based nanocarrier	Tumor hypoxia	Doxorubicin	Improved hypoxia induced chemoresistance reversal	[155]
Block copolymer nanoparticles	Tumor altered pH	Cisplatin, F3 peptide	Rapid tumor regression, avascular effect with significant vascular necrosis	[156]
Gold nanoparticles	Tumor altered pH	Doxorubicin	Elevated apoptosis, enhanced toxicity	[157]
Chitosan nanoparticles	Tumor altered pH	Mesoporous silica nanoparticles	Increased solubility and improved anticancer properties	[158]
Poly(L-histidine) (PHIS) and hyaluronic acid nanoparticles	Tumor altered pH	Doxorubicin, Anti-tumor immune regulator (R848)	Dual pH responsive nanoparticles, excellent tumor-targeting ability, inhibition of tumor growth	[159]
Multifunctional co block polymers-based nanosystems	Tumor altered pH	Doxorubicin, lectin	8-fold higher toxicity than free drug, 100% osteosarcoma cell death	[160]
Polyamidoamine (PAMAM) dendrimers	Tumor altered pH	Platinum-prodrug	pH-triggered size switching, improved drug penetration and therapeutic efficacy	[161]
Calcium carbonate aragonite nanocrystal	Tumor altered pH	Doxorubicin	Higher uptake of pH sensitive nanocrystals with great reduction of tumor growth	[162]
Micellar cationic lipid-assisted polymeric nanoparticles	Tumor altered pH	siRNA, Antibody of programmed cell death protein 1 (PD-1)	Neutralization of the tumor pH, significant inhibition of tumor growth	[163]
Magnetic nanoparticles	Alteration of metabolic pathways	Glucose	Enhanced internalization of glucose coated nanoparticles	[164]
Bis-2-(5-phenylacetamido-1,2,4-thiadiazol-2-yl) ethyl sulfide (BPTES) nanoparticles	Alteration of metabolic pathways	Glutaminase inhibitor (CB-839), metformin	Effective inhibition of glutaminase, reduced tumor growth	[165]
Gold nanoparticles	Alteration of metabolic pathways	3-bromopyruvate (3-BP)	Enhanced ability to modulate cancer cell metabolism, mediating	[166]
Mesoporous silica nanoparticles	Tumor ECM modulation	Collagenase nanocapsules	Enhanced nanocarrier penetration, improved therapeutic efficiency	[167]
Liposome-based nanoparticles	Tumor ECM modulation	Collagenase, paclitaxel	Improved drug penetration, degradation of ECM correlated to reduction in metastasis	[168]

**Table 4:** A list of preclinical studies using siRNA-based delivery systems for reduction in tumor growth, vascularization, metastasis, and chemotherapeutic resistance.

Type of Nanoparticles	Target Gene/Protein	Target Areas	Reference
Layer by layer nanoparticles	MDR1	Chemotherapeutics resistance	[212]
PEG2000-PE PM	Survivin	Chemotherapeutics resistance	[213]
Nanocopolymer	Survivin	Chemotherapeutics resistance	[214]
Liposomal nanoparticles	FOXM1	Cell growth and progression of cell cycle	[215]
Polymer-lipid nanoparticles	VEGF	Cell growth and progression of cell cycle	[216]
PEG-modified lipid nanoparticles	Transferrin	Cell growth and progression of cell cycle	[217]
PEG-modified lipid nanoparticles	EpCAM	Cell growth and progression of cell cycle	[218]
PEI-modified gold nanoparticles	eEF2K	Cell growth and progression of cell cycle	[219]
Lipid nanoparticles	BCR-ABL fusion gene	Cell growth and progression of cell cycle	[220]
Agarose gel nanoparticles	POLR2A	Cell growth and progression of cell cycle	[221]
Mesoporous silica nanoparticles	PLK1	Cell growth and progression of cell cycle	[222]
Silica-nanoparticles	mTORC2	Cell growth and progression of cell cycle	[223]
Fab’s antibody modified LNP	HB-EGF	Cell growth and progression of cell cycle	[224]
Lipid-dendrimer-calcium-phosphate nanoparticles	PD-L1	Cell growth and progression of cell cycle	[225]
Chitosan nanoplexes	VEGF-A, VEGFR-1, VEGFR-2 and neuropilin-1	Angiogenesis and Tumor Microenvironment	[226]
ICAM-1 conjugated liposomes	Lipocalin 2	Angiogenesis and Tumor Microenvironment	[227]
RGD-PEG-ECO nanoparticles	DANCR	Tumor invasion and metastasis	[228]
CoFe-nanoparticles	EF2K	Tumor invasion and metastasis	[229]

Abbreviations: FOXM1: Fork head box protein M1, PEI: Polyethylimine, eEF2K: Eukaryotic Elongation Factor 2 Kinase, POLR2A (RNA Polymerase II Subunit A), PLK1: Polo-like kinase 1, mTORC2: Mammalian target of rapamycin complex 2, LNP: Lipid nanoparticles, HB-EGF: Heparin-binding EGF-like growth factor, VEGF: Vascular endothelial growth factors, VEGFR: Vascular endothelial growth factor receptors, ICAM-1: Intercellular Adhesion Molecule 1, MDR1: MDR gene 1, PEG2000-PE: Polyethylene glycol2000-phosphatidyl ethanolamine, PM: Polymeric micelles, DANCR: Differentiation Antagonizing Non-Coding RNA; eEF2K: Eukaryotic Elongation Factor 2 Kinase, CO-Fe: Cobalt-ferric, PEG: Polyethylene glycol, EpCAM: Epithelial cell adhesion molecule, BCR-ABL: breakpoint cluster region-Abelson.

**Table 5:** Representative list of anticancer siRNA-mediated nanoparticles in clinical trials.

Therapeutic Name	Delivery System	Type of Cancer	Status	Reference
NBF-006	Lipid nanoparticles	Non-small cell lung carcinoma, pancreatic carcinoma, colorectal carcinoma	Phase I/recruiting	[237]
siRNA-EphA2- DOPC	Lipid nanoparticles	Advanced cancers	Phase I/Not completed yet	[238]
ALN-VSP02	Lipid nanoparticles	Solid liver tumors	Phase I/Completed	[239]
siG12D LODER	LODER polymer	Pancreatic cancer, pancreatic ductal Adenocarcinoma	Phase II/Ongoing	[240]
Atu027	Lipid nanoparticles	Metastatic pancreatic cancer (II), solid tumors (I)	Phase II/Completed	[241]
TKM- PLK1 (TKM-080301)	Lipid nanoparticles	Hepatocellular carcinoma (II), adrenal cortical carcinoma (II), neuroendocrine tumor (II), solid tumors (I)	Phase II/Completed	[209]

## Data Availability

All data generated and analyzed during this study are included in this article.
